# The broad host range pathogen *Sclerotinia sclerotiorum* produces multiple effector proteins that induce host cell death intracellularly

**DOI:** 10.1111/mpp.13333

**Published:** 2023-04-10

**Authors:** Toby E. Newman, Haseong Kim, Yuphin Khentry, Kee Hoon Sohn, Mark C. Derbyshire, Lars G. Kamphuis

**Affiliations:** ^1^ Centre for Crop and Disease Management, School of Molecular and Life Sciences Curtin University Bentley Western Australia Australia; ^2^ Plant Immunity Research Center Seoul National University Seoul 08826 Republic of Korea; ^3^ Department of Agricultural Biotechnology Seoul National University Seoul 08826 Republic of Korea; ^4^ Research Institute of Agriculture and Life Sciences Seoul National University Seoul 08826 Republic of Korea

**Keywords:** effector, localization, necrotrophic fungus, NLR protein, plant pathogen, RxLR motif, *Sclerotinia sclerotiorum* (white mould)

## Abstract

*Sclerotinia sclerotiorum* is a broad host range necrotrophic fungal pathogen, which causes disease on many economically important crop species. *S. sclerotiorum* has been shown to secrete small effector proteins to kill host cells and acquire nutrients. We set out to discover novel necrosis‐inducing effectors and characterize their activity using transient expression in *Nicotiana benthamiana* leaves. Five intracellular necrosis‐inducing effectors were identified with differing host subcellular localization patterns, which were named intracellular necrosis‐inducing effector 1–5 (SsINE1–5). We show for the first time a broad host range pathogen effector, SsINE1, that uses an RxLR‐like motif to enter host cells. Furthermore, we provide preliminary evidence that SsINE5 induces necrosis via an NLR protein. All five of the identified effectors are highly conserved in globally sourced *S. sclerotiorum* isolates. Taken together, these results advance our understanding of the virulence mechanisms employed by *S. sclerotiorum* and reveal potential avenues for enhancing genetic resistance to this damaging fungal pathogen.

## Graphical abstract

Five effectors produced by *Sclerotinia sclerotiorum* induce necrosis intracellularly. One of these uses an RxLR‐like motif to enter host cells and another requires a host NLR gene to activate robust cell death.

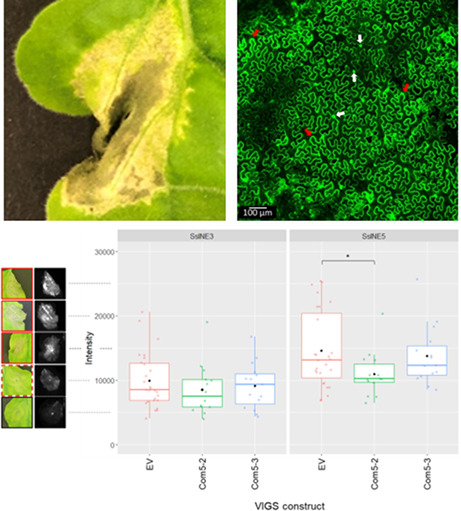

## 1 INTRODUCTION

*Sclerotinia sclerotiorum* is a fungus that infects hundreds of mainly dicotyledonous plant species (Boland & Hall, 1994; Derbyshire et al., 2022). It causes disease on many economically important crop species, including soybean (*Glycine max*), oilseed rape (*Brassica napus*), and pulses, resulting in significant economic damage (Antwi‐Boasiako et al., 2021; Saharan & Mehta, 2008; Zheng et al., 2020). Therefore, it is important to understand the mechanistic basis of disease promotion by *S. sclerotiorum* to develop strategies towards controlling it.

*S. sclerotiorum* is generally considered necrotrophic, as it kills host cells and derives nutrients from the resulting dead tissue. However, recent evidence has indicated that *S. sclerotiorum* has a transient biotrophic phase as it colonizes host tissue (Kabbage et al., 2015; Liang & Rollins, 2018). *S. sclerotiorum* secretes the well‐characterized molecule oxalic acid, which has diverse roles in pathogenesis including guard cell deregulation and oxidative burst manipulation (Cessna et al., 2000; Guimaraes & Stotz, 2004; Williams et al., 2011). *S. sclerotiorum* also produces an array of cell wall‐degrading enzymes, which macerate host tissue and facilitate penetration by fungal hyphae (Riou et al., 1991). Host tissue acidification by oxalic acid and other acids produced by *S. sclerotiorum* enhances both the expression and activity of cell wall‐degrading enzymes, further promoting disease progression (Cotton et al., 2003; Favaron et al., 2004; Rollins & Dickman, 2001).

*S. sclerotiorum* also secretes several characterized proteinaceous effectors, many of which elicit necrosis in host tissue. These include two necrosis‐ and ethylene‐inducing peptides (SsNEP1 and SsNEP2); an EF‐hand motif‐containing protein (Ss‐Caf1); the small, secreted, cysteine‐rich proteins SsSSVP1 and SsNE1–6; and the cerato‐platanin protein SsCP1 (Dallal Bashi et al., 2010; Lyu et al., 2016; Seifbarghi et al., 2020; Xiao et al., 2014; Yang et al., 2018, 2022). SsNEP1, SsNEP2, and SsNE1–5 function extracellularly in the host apoplast, whereas Ss‐Caf1, SsSSVP1, and SsNE6 function inside host cells (Dallal Bashi et al., 2010; Lyu et al., 2016; Seifbarghi et al., 2020; Xiao et al., 2014). SsCP1 induces cell death intracellularly but induces a stronger cell death response when secreted, indicating that it activates necrosis both intra‐ and extracellularly (Yang et al., 2018).

It is not well understood how fungal effectors enter host cells to localize to their intracellular virulence targets. SsSSVP1 was shown to translocate into host cells and move from cell to cell in the absence of *S. sclerotiorum*; however, the mechanism underlying this movement remains unknown (Lyu et al., 2016). Cell‐to‐cell movement has been shown previously in two *Magnaporthe oryzae* effectors (Khang et al., 2010). Intracellular oomycete effectors tend to possess two N‐terminal motifs, RxLR (Arg‐Xaa‐Leu‐Arg) and dEER (Asp‐Glu‐Glu‐Arg), that mediate host cell entry (Bouwmeester et al., 2011). A common host cell entry motif has not been identified in intracellular fungal effectors; however, an RxLR‐like motif has been found to be involved in host cell entry in some fungal plant pathogen effectors including *Melampsora lini* AvrL567 and AvrM, *Fusarium oxysporum* f. sp. *lycopersici* Avr2, and *Leptosphaeria maculans* AvrLm6 (Kale, 2012; Kale et al., 2010; Rafiqi et al., 2010). An RxLR‐like motif has also been identified in the MiSSP7 protein secreted by the mutualistic ectomycorrhizal fungus *Laccaria bicolor*, which is required for entry into root cells and symbiosis development (Plett et al., 2011). Thus far, no functional RxLR‐like motif has been described in a broad host range plant pathogen.

Intra‐ and extracellular immune receptors monitor host cells to detect pathogen invasion. Intracellular immune receptors are typically nucleotide‐binding leucine‐rich repeat (NLR) proteins that directly or indirectly recognize the presence of pathogen effectors in what is known as a gene‐for‐gene interaction (Flor, 1956; Jones & Dangl, 2006). Effector recognition results in activation of effector‐triggered immunity, which often culminates in localized cell death termed the hypersensitive response (HR). Detection of a biotrophic or hemibiotrophic effector results in immunity. On the other hand, detection of a necrotrophic effector and activation of an HR results in susceptibility in an inverse gene‐for‐gene manner, as the pathogen derives nutrients from the dead tissue (Liu et al., 2006; Lorang et al., 2007). Inverse gene‐for‐gene interactions have been described in the interaction between narrow host range necrotrophic fungal pathogens and host species (Liu et al., 2017; Shao et al., 2021). Whether *S. sclerotiorum* uses effectors to hijack NLR‐mediated HR is currently unknown, although the finding that an *Arabidopsis thaliana* NLR contributes to *S. sclerotiorum* susceptibility suggests that this may occur during infection (Barbacci et al., 2020).

In a previous bioinformatic study, 70 putative effector genes were predicted in the *S. sclerotiorum* genome (Derbyshire et al., 2017). In this study, we show that five of these putative effectors trigger necrosis in planta. We demonstrate that the effectors function intracellularly and localize to different subcellular compartments. For the first time, we show evidence that a broad host range pathogen effector, *S. sclerotiorum* intracellular necrosis‐inducing effector 1 (SsINE1), enters host cells using an RxLR‐like motif. A gene knockdown screen in *Nicotiana benthamiana* indicated that SsINE5 may induce host cell death via an NLR protein, a novel mechanism of cell death induction for a broad host range pathogen. Collectively, these results further our understanding of the molecular mechanisms underlying virulence of a broad host range fungal pathogen.

## 2 RESULTS

### 2.1 Multiple *S. sclerotiorum* putative effectors act intracellularly to induce necrosis in planta

We aimed to discover novel *S. sclerotiorum* necrosis‐inducing effectors. To this end, a set of 70 putative *S. sclerotiorum* effectors previously identified in the reference isolate 1980 were selected as initial candidates to screen for necrosis‐inducing effectors (Derbyshire et al., 2017). All 70 putative effectors were found to be conserved in the aggressive Australian isolate CU8.24 (Denton‐Giles et al., 2018). The localization of the putative CU8.24 effectors was predicted using ApoplastP (Sperschneider et al., 2018). This revealed 46 putative apoplastic effectors and 24 putative cytoplasmic effectors, the latter of which are predicted to enter host cells during infection. One of the 24 putative cytoplasmic effectors was found to be a conserved GTP‐binding protein, SarA, involved in membrane trafficking, and was therefore removed from further analysis. Several characterized *S. sclerotiorum* effectors have been shown to function inside host cells and we hypothesized that there are additional as yet uncharacterized effectors that function intracellularly to induce host cell death. Therefore, we set out to screen the 23 putative cytoplasmic effectors for necrosis‐inducing activity in planta. Additionally, there were five putative apoplastic effectors that have not been screened for necrosis‐inducing activity in other studies and were significantly up‐regulated during infection of *B. napus*, which were also included in the assay.

**FIGURE 1:**
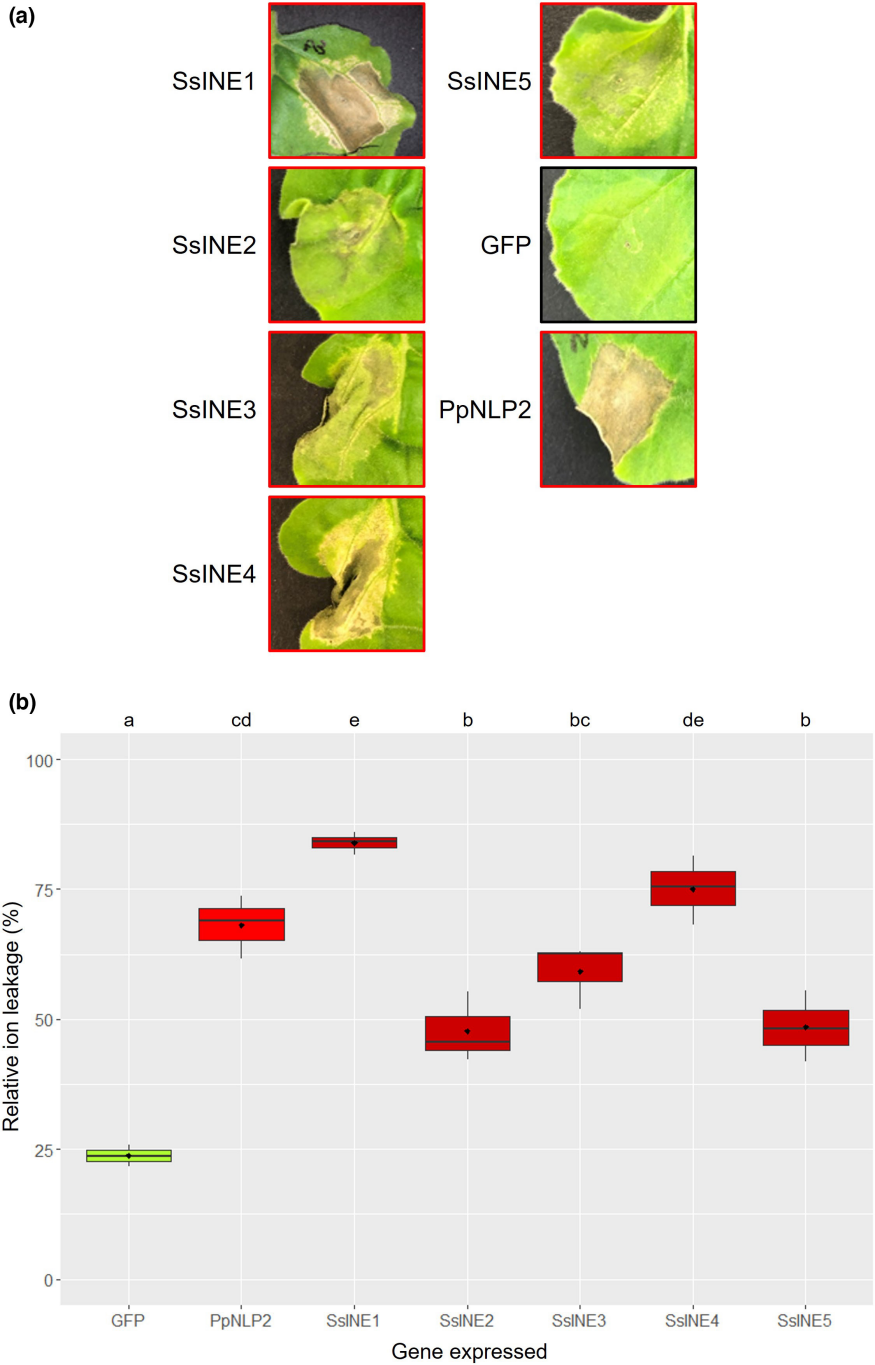
Necrosis‐inducing activity of SsINE proteins in *Nicotiana benthamiana* leaf tissue. (a) Macroscopic cell death symptoms induced by agroinfiltration of mature effector proteins SsINE1–5. Green fluorescent protein (GFP) was included as a negative control. PpNLP2 was included as a positive control. Photographs were taken at 7 days postinfiltration. A red border indicates cell death symptoms; a black border indicates no cell death symptoms. (b) Relative ion leakage induced by agroinfiltration of SsINE1–5. GFP was included as a negative control. PpNLP2 was included as a positive control. Different letters above the boxes indicate significant differences, as determined by analysis of variance with a post hoc Tukey honestly significant difference test.

Seventeen putative cytoplasmic and four putative apoplastic effectors were successfully cloned, tagged at the C‐terminus with green fluorescent protein (GFP), and expressed in *N. benthamiana* leaves by *Agrobacterium*‐mediated transient expression (agroinfiltration) with appropriate controls (Table 1). As expected, the negative control of GFP with a C‐terminal 6×His‐tag (GFP‐his) induced no cell death and the positive control, *Peyronellaea pinodes* NLP2 expressed with the *Medicago truncatula* PR‐1 signal peptide (SP) and a C‐terminal 6×His‐tag (SP‐NLP2‐his), induced strong necrosis (Debler et al., 2021). The putative cytoplasmic effectors were expressed without their predicted native SP to retain the mature effector proteins inside host cells. Four of these effectors consistently induced necrosis. Necrosis symptoms became visible from 3 days postinfiltration (DPI) and photographs were taken at 7 DPI. These are hereafter referred to as *S. sclerotiorum* intracellular necrosis‐inducing effector 1–4 (SsINE1–4) (Figure 1, Table 1). When expressed with the MtPR‐1 SP to export the effector proteins to the apoplast, SsINE1 and SsINE2 induced an attenuated cell death response, whereas SsINE3 and SsINE4 induced no macroscopic cell death symptoms, indicating that the effectors require cytoplasmic localization for full activity (Figure S1). The mature sequences of the four putative apoplastic effectors were expressed with the MtPR‐1 SP to export the effector proteins to the apoplast and assay for cell death symptoms. One of the effectors induced weak necrosis. We expressed this effector without the MtPR‐1 SP and surprisingly observed a stronger cell death response. This effector is hereafter referred to as SsINE5 (Figures 1 and S1, Table 1). Western blot analyses confirmed accumulation of the SsINE‐GFP fusion proteins with and without an SP except for SP‐SsINE4‐GFP, which did not elicit necrosis (Figure S3).

**TABLE 1:** Putative *Sclerotinia sclerotiorum* effectors.

Putative effector	ApoplastP prediction	LOCALIZER v. 1.0.4 prediction	WoLF PSORT prediction	Mature effector length (amino acids)	Up‐regulated during infection	Necrosis
*Sscle01g005390*	Cytoplasmic	–	Chloroplastic	102	Yes (3)	No
*Sscle01g006330*	Apoplastic	–	NA	121	Yes (1)	No
*Sscle01g009960*	Cytoplasmic	mTP	Chloroplastic, cytoplasmic	180	No	No
*Sscle02g012940* (*SsINE1*)	Cytoplasmic	–	Nuclear	54	Yes (1, 2)	Yes
*Sscle02g021780*	Cytoplasmic	–	Cytoplasmic	186	No	No
*Sscle04g039210*	Cytoplasmic	–	Cytoplasmic	112	No	No
*Sscle05g045060* (*SsINE5*)	Apoplastic	–	Mitochondrial	73	Yes (1, 2)	Yes
*Sscle05g046060* (*SsINE2*)	Cytoplasmic	–	Mitochondrial	68	Yes (3, 5)	Yes
*Sscle05g046070*	Cytoplasmic	NLS	Chloroplastic	194	No	No
*Sscle06g050820*	Cytoplasmic	–	Chloroplastic	63	Yes (2)	No
*Sscle07g057000*	Cytoplasmic	mTP	Chloroplastic	173	No	No
*Sscle10g075140*	Apoplastic	–	NA	69	Yes (1, 2)	No
*Sscle10g080580*	Cytoplasmic	–	Cytoplasmic	55	No	No
*Sscle11g081020*	Cytoplasmic	–	–	34	No	No
*Sscle12g087960* (*SsINE3*)	Cytoplasmic	–	Chloroplastic	138	Yes (4)	Yes
*Sscle12g088660*	Cytoplasmic	–	Chloroplastic	185	No	No
*Sscle13g094760* (*SsINE4*)	Cytoplasmic	–	–	125	No	Yes
*Sscle13g094920*	Cytoplasmic	–	Chloroplastic, cytoplasmic	182	No	No
*Sscle13g095230*	Cytoplasmic	NLS	Nuclear	164	No	No
*Sscle14g100310*	Apoplastic	–	NA	268	Yes (1, 2)	No
*Sscle16g107890*	Cytoplasmic	–	Mitochondrial, nuclear	90	Yes (2)	No

*Note*: ^1^Derbyshire et al. (2017); ^2^Westrick et al. (2019); ^3^Seifbarghi et al. (2020); ^4^Mwape et al. (2021); ^5^Kusch et al. (2022).

To validate and quantify the macroscopic cell death symptoms caused by the intracellular effector proteins, we conducted an ion leakage assay. As expected, PpNLP2 induced leakage of significantly more ions from the agroinfiltrated leaf section than GFP, as would be expected in necrotic tissue. The ion leakage results corroborated the visual cell death symptoms in that all SsINEs induced greater ion leakage than GFP. Furthermore, SsINE1 and SsINE4 were the most potent necrosis‐inducing effectors, as their expression resulted in greater ion leakage than that caused by the other SsINEs (Figure 1b). In summary, five necrosis‐inducing effectors were identified that function intracellularly.

### 2.2 The necrosis‐inducing effectors localize to different subcellular compartments in host cells

Fungal pathogen effectors are known to function in various subcellular compartments. To provide insight into the cellular targets of the putative effectors, we predicted their subcellular localization using in silico methods and used confocal microscopy to determine the subcellular localization of the SsINEs in *N. benthamiana* epidermal cells. LOCALIZER did not predict any transit peptides or nuclear localization signals in the SsINEs. However, two of the nonnecrosis‐inducing effectors were found to harbour putative mitochondrial transit peptides and two others were found to harbour putative nuclear localization signals (Table 1). WoLF PSORT predicted chloroplastic, cytoplasmic, mitochondrial, and nuclear localization of the effector candidates screened. SsINE1 was predicted to have nuclear localization, SsINE2 and SsINE5 were predicted to have mitochondrial localization, and SsINE3 was predicted to have chloroplastic localization (Table 1). A prediction was not made for SsINE4.

**FIGURE 2:**
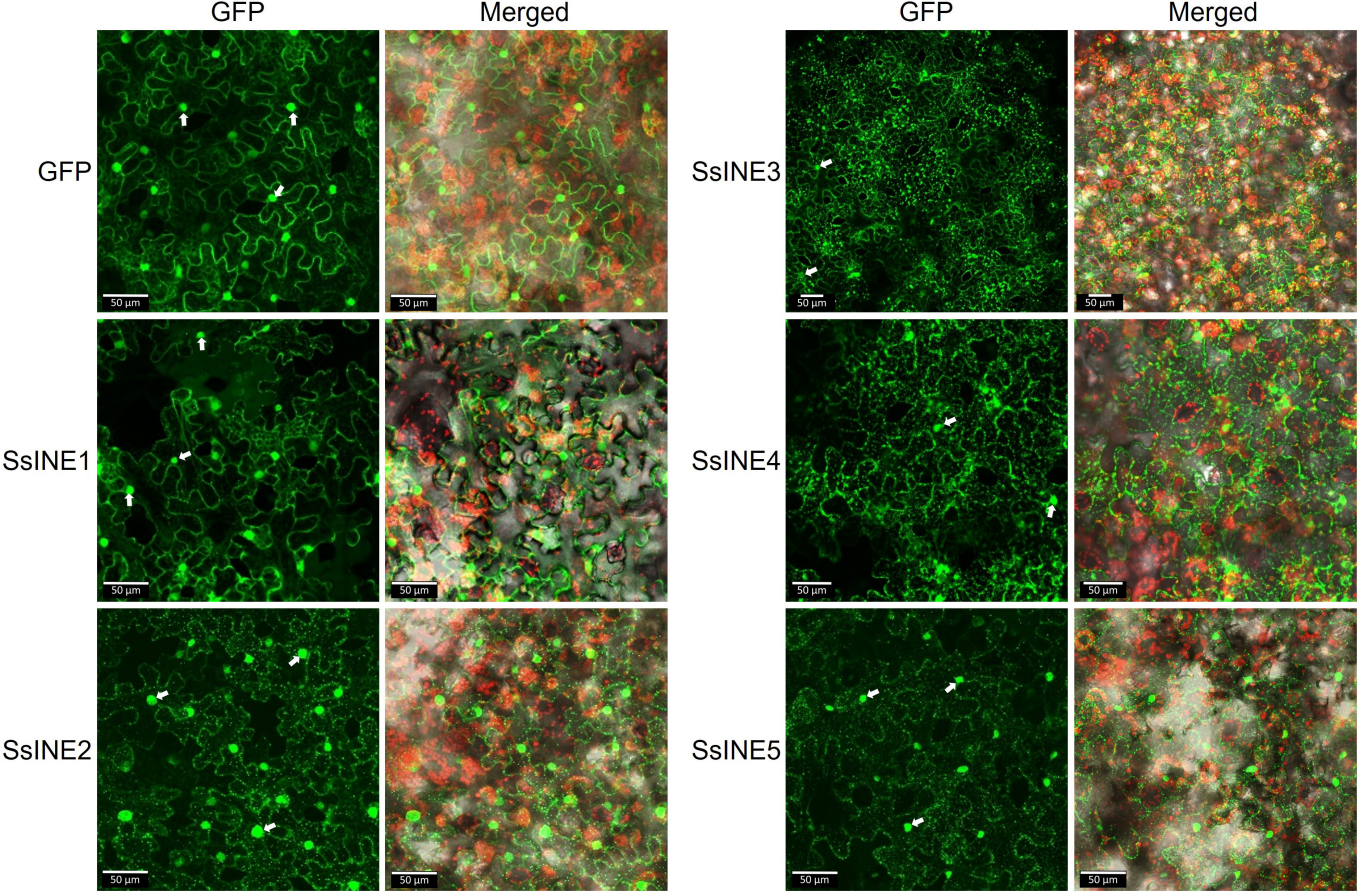
Subcellular localization of SsINE proteins expressed without a signal peptide in *Nicotiana benthamiana* epidermal cells. Panels on the left‐hand side labelled “GFP” show green fluorescent protein (GFP) fluorescence. Panels on the right‐hand side labelled “Merged” show GFP fluorescence, chloroplast autofluorescence, and bright field images. The white arrows indicate nuclei. Images are *z*‐projections. The scale bars are 50 μm.

Following on from the in silico localization prediction, the localization of the GFP‐tagged SsINE proteins in *N. benthamiana* epidermal cells was investigated using confocal microscopy. As expected, GFP exhibited nucleocytoplasmic distribution. SsINE1 was also observed in the nuclei and cytoplasm. SsINE2 and SsINE5 displayed similar localization patterns, localizing to both nuclei and punctate structures within the cells. The size of the punctae is consistent with that of mitochondria, although without mitochondrial marker colocalization we cannot draw any firm conclusions (Jaipargas et al., 2015). The visualization of chloroplast autofluorescence enabled the observation that SsINE3 appeared to cluster primarily around chloroplasts. SsINE4 primarily localized to punctate structures within the cells. SsINE3 and SsINE4 also partially localized to nuclei (Figure 2). When expressed with the MtPR‐1 SP, apoplastic localization of the effectors could be observed, demonstrating that the SP directed the proteins for export. Interestingly, GFP fluorescence could also be observed within nuclei of host cells when the SsINE‐GFP variants were expressed with an SP (Figure S2). Collectively, these data indicate that the SsINEs target different subcellular compartments to induce cell death.

### 2.3 The necrosis‐inducing effectors are highly conserved in globally sourced *S. sclerotiorum* isolates

As previously mentioned, the North American *S. sclerotiorum* reference isolate 1980 and Australian isolate CU8.24 both harbour all five SsINEs. We sought to investigate the conservation of the five SsINEs in *S. sclerotiorum* isolates found across the world and on diverse host species to provide some insight into their dispensability for *S. sclerotiorum* fitness. This analysis was conducted using genome sequences of 26 *S. sclerotiorum* isolates, including 1980 and CU8.24 (Derbyshire et al., 2017, 2019).

**FIGURE 3:**
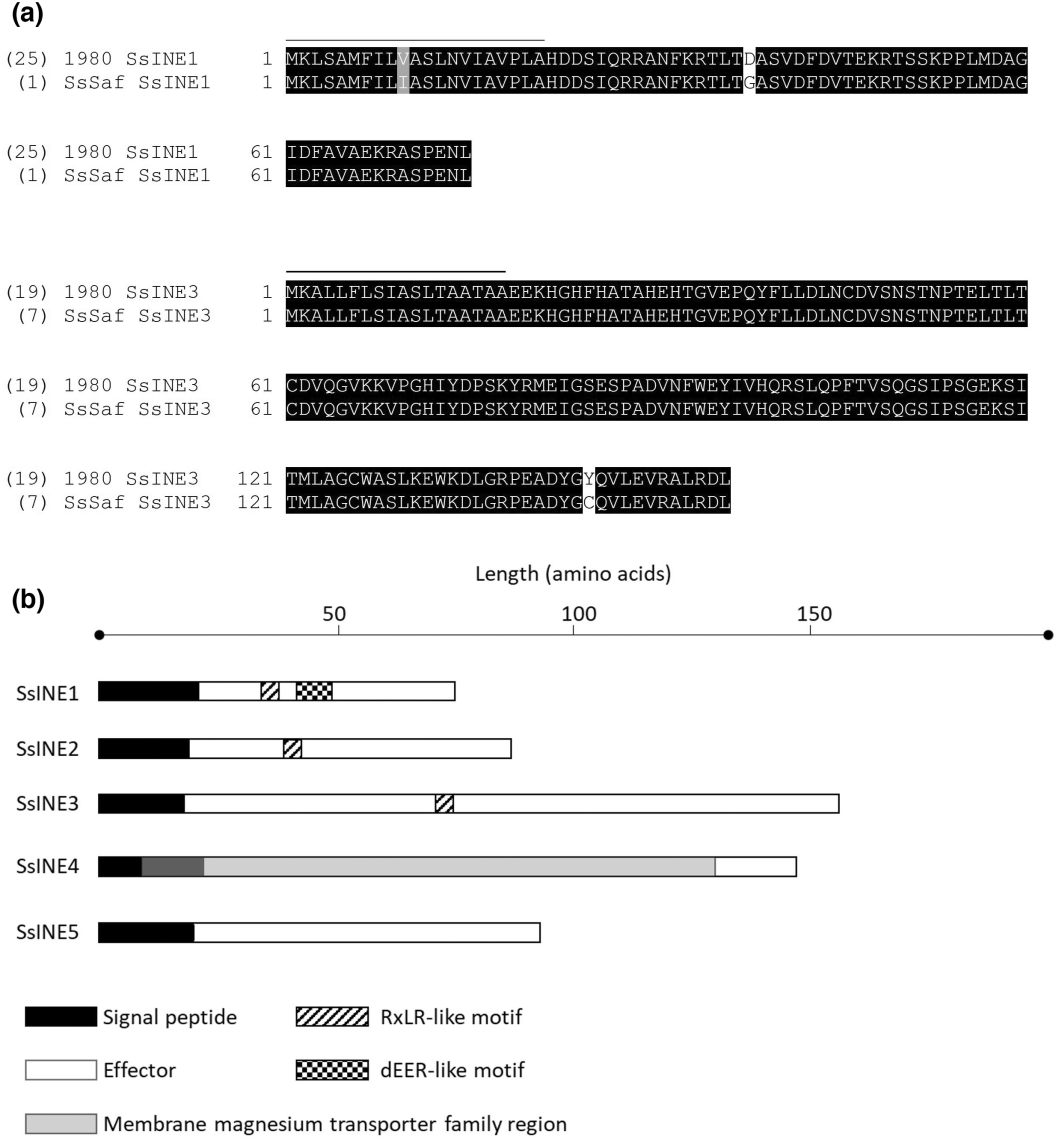
Amino acid conservation and predicted features of SsINEs. (a) Alignment of two alleles of SsINE1 and SsINE3 within 26 globally sourced *Sclerotinia sclerotiorum* isolates. The number in brackets indicates the number of isolates that harbour the corresponding allele. The line above the amino acids shows the predicted signal peptide sequence. (b) Length and predicted features of SsINE proteins. Predicted domains and motifs are represented by the different shades and patterns.

The SsINEs were present in all 26 isolates. Furthermore, the amino acid conservation was remarkably high. The amino acid sequences of SsINE2, SsINE4, and SsINE5 were identical in all isolates. The amino acid sequence of SsINE1 was identical in 25 isolates; however, an isolate from South Africa (Sssaf) had one amino acid polymorphism in the predicted SP (V10I) and another amino acid polymorphism in the mature effector protein (D38G). The amino acid sequence of SsINE3 was identical in 19 isolates, with the other seven isolates harbouring only one amino acid polymorphism in the mature effector protein (Y145C). These seven isolates comprised the South African isolate Sssaf and six isolates sourced from Australia (Figure 3a, Table S1). To summarize, the amino acid sequences of the SsINEs are highly conserved.

### 2.4 An RxLR‐like motif facilitates translocation of the SsINE1 effector protein into host cells

**FIGURE 4:**
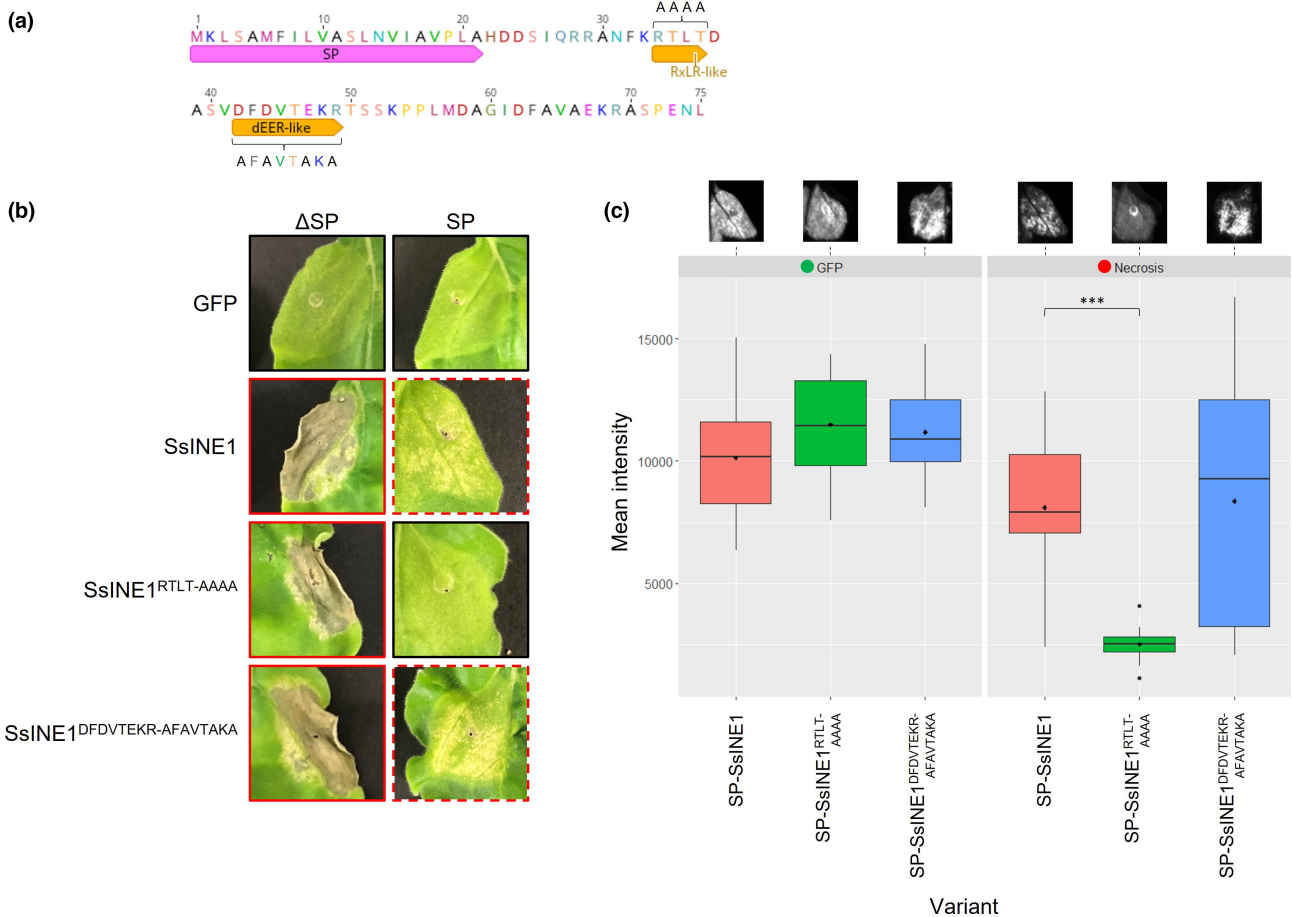
Investigation into the RxLR‐like and dEER‐like motifs of SsINE1. (a) Amino acid sequence of the CU8.24 SsINE1 protein. The SP annotation indicates the predicted signal peptide. Amino acid letters above the RxLR‐like and dEER‐like motifs indicate the sequence of the mutated variants (RTLT‐AAAA and DFDVTEKR‐AFAVTAKA). (b) Macroscopic cell death symptoms induced by agroinfiltration of SsINE1 variants with and without a signal peptide. Green fluorescent protein (GFP) was included as a negative control. A red border indicates cell death symptoms; a dashed red border indicates attenuated cell death; a black border indicates no cell death symptoms. (c) The left and right panels are boxplots showing GFP fluorescence and red light fluorescence, respectively, of agroinfiltrated *Nicotiana benthamiana* leaf sections. Representative fluorescence images of the “SP” leaves in panel (b) are shown above the boxplot. Black diamonds represent the mean values. The three asterisks (***) indicate a significant difference from SP‐SsINE1 (*p* ≤ 0.001), as determined by a Student's *t* test.

To investigate the possible functions of the SsINEs, they were scanned for protein domains using InterProScan. Besides the predicted SPs, this scan revealed a membrane magnesium transporter family entry in SsINE4 that partially overlapped with the predicted SP (Figure 3b). Intracellular oomycete effectors often harbour RxLR/RxL and dEER translocation motifs, and some intracellular fungal effectors harbour RxLR‐like motifs (Kale, 2012; Links et al., 2011). Manual scanning of the amino acid sequences for RxLR/RxL and dEER motifs revealed an RxLR‐like (RTLT) and a dEER‐like motif (DFDVTEKR) in the N‐terminus of the mature SsINE1 protein (Figures 3b and 4a). Further investigation into the presence of an expanded RxLR‐like [R/K/H]x[L/M/I/F/Y/W]x motif, as defined by Kale et al. (2010), in the N‐termini of the mature candidate effectors that were screened herein revealed an RxLR‐like motif in SsINE2 (HKIC), SsINE3 (HIYD), and 11 out of 16 of the nonnecrosis‐inducing effectors. Six of the nonnecrosis‐inducing effectors harbour two RxLR‐like motifs (Table S2). We functionally characterized the SsINE1 RxLR‐like and dEER‐like motifs due to the clear cell death symptoms when SsINE1 was expressed with an SP, indicating translocation into host cells (Figure S1). Wet laboratory validation of the RxLR‐like motifs identified in SsINE2, SsINE3, and the 11 nonnecrosis‐inducing effectors would be required to determine their function, although the absence of cell death induced by SP‐SsINE3 suggests that the SsINE3 RxLR‐like motif does not mediate host cell entry.

We hypothesized that the SsINE1 motifs may be involved in translocation of the effector into host cells after secretion by *S. sclerotiorum* during infection. To investigate whether the motifs are involved in host cell translocation, we used site‐directed mutagenesis to mutate the motif residues to alanine residues (RTLT‐AAAA and DFDVTEKR‐AFAVTAKA, respectively) (Figure 4a). The induced SsINE1 variants were C‐terminally tagged with GFP and then assessed for necrosis‐inducing activity and localization by agroinfiltration in *N. benthamiana* leaves. When the mutated variants were expressed without an SP, they induced strong cell death symptoms comparable to wild‐type SsINE1 (Figure 4b). This result demonstrates that the motifs are not required for effector activity.

The SsINE1‐GFP variants were fused with MtPR‐1 SP at the N‐terminus. SP‐SsINE1^RTLT‐AAAA^‐GFP induced no visible cell death symptoms, whereas SP‐SsINE1^DFDVTEKR‐AFAVTAKA^‐GFP induced necrosis comparable to the wild type (Figure 4b). Additionally, we used red light fluorescence to quantify and compare the cell death symptoms caused by the SsINE1 variants expressed with SPs. When excited with green light, leaf tissue undergoing cell death emits red light due to chlorophyll fluorescence as a result of thylakoid membrane disassembly (Landeo Villanueva et al., 2021). To corroborate the macroscopic cell death symptoms, we found that SP‐SsINE1^RTLT‐AAAA^‐GFP induced significantly less cell death than the wild‐type variant, as demonstrated by the reduced red light fluorescence when agroinfiltrated zones were excited with green light. On the other hand, there was no significant difference between red light fluorescence induced by SP‐SsINE1^DFDVTEKR‐AFAVTAKA^‐GFP and SP‐SsINE1‐GFP (Figure 4c). These results indicate that the RxLR‐like motif, but not the dEER‐like motif, is required for cell death induction when SsINE1 is expressed with an SP.

To test if the reduction in cell death symptoms was caused by reduced expression or accumulation of the SP‐SsINE1^RTLT‐AAAA^‐GFP variant, we also measured GFP expression. There was no significant difference in GFP expression between the SsINE1 variants, indicating that the reduction in cell death symptoms was not a result of reduced accumulation of the protein (Figure 4c). This finding was confirmed by western blot analysis using an anti‐GFP antibody. To clarify, a reduction in protein expression was not observed when SP‐SsINE1^RTLT‐AAAA^‐GFP was expressed relative to SP‐SsINE1‐GFP (Figure S4). All induced variant proteins were detected by immunoblotting. Interestingly, an additional higher‐molecular‐weight band was detected when SsINE1 variants were expressed with an SP, which could be unprocessed effector proteins carrying the SP or posttranslationally modified proteins (Figure S4).

**FIGURE 5:**
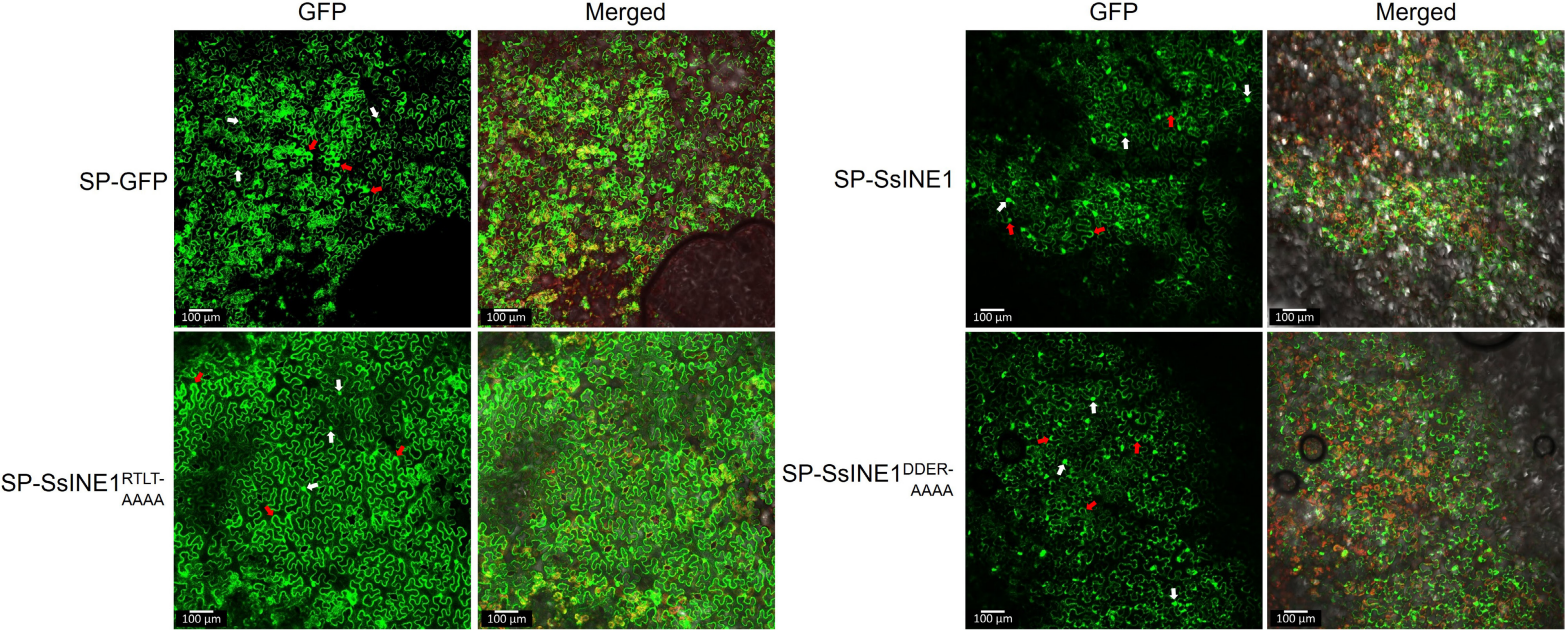
Subcellular localization of SsINE1 variants expressed with a signal peptide (SP) in *Nicotiana benthamiana* epidermal cells. Green fluorescent protein (GFP) was included as a control. The leaf samples were plasmolysed prior to mounting on microscope slides. Panels on the left‐hand side labelled “GFP” show GFP fluorescence. Panels on the right‐hand side labelled “Merged” show GFP fluorescence, chloroplast autofluorescence, and bright field images. The white arrows indicate nuclei; the red arrows indicate apoplastic localization. Images are *z*‐projections. The scale bars are 100 μm.

Confocal microscopy was used to investigate whether the RxLR‐like motif is involved in translocation of SsINE1 into *N. benthamiana* epidermal cells after export into the apoplast. GFP and GFP‐tagged SsINE1 variants were expressed without an SP and all displayed similar nucleocytoplasmic localization (Figure S5). When GFP was expressed with an SP, fluorescence was detected in the apoplast (Figure 5). Cytosolic and nuclear localization was also apparent, possibly as a result of escape from the saturated secretory pathway caused by *Agrobacterium*‐mediated overexpression. When SP‐SsINE1‐GFP was expressed, the predominant localization observed was in the host cell nuclei, although some fluorescence was also observed in the apoplast. A similar localization pattern was observed for SP‐SsINE1^DFDVTEKR‐AFAVTAKA^‐GFP. However, when the localization of SP‐SsINE1^RTLT‐AAAA^‐GFP was analysed, it was apparent that the protein was predominantly localized to the apoplastic space (Figure 5). The fluorescence signal in the apoplast was clearer for SP‐SsINE1^RTLT‐AAAA^‐GFP than for SP‐GFP. The SsINE1^RTLT‐AAAA^‐GFP fusion protein may be more stable than GFP alone in the acidic and protease‐rich apoplast. These confocal microscopy results support the hypothesis that the RxLR‐like motif facilitates entry into host cells and provide an understanding of the reduction in cell death symptoms induced by SP‐SsINE1^RTLT‐AAAA^‐GFP. Taken together, these results show that the SsINE1 effector uses an RxLR‐like motif to enter host cells, where it induces cell death.

### 2.5 SsINE5 may induce cell death in an NLR‐dependent manner

Many effector proteins that induce host cell death intracellularly are recognized by immune receptors, which in turn activate programmed cell death. We sought to investigate whether any of the SsINEs induce cell death that is dependent on a host intracellular immune receptor of the NLR type. To this end, a virus‐induced gene silencing (VIGS) approach was used to posttranscriptionally silence the expression of the repertoire of predicted NLR genes in the *N. benthamiana* genome. The *N. benthamiana* NLR (NbNLR) VIGS library was generated in Ahn et al. (2022). Subsequently, impairment of SsINE‐induced cell death was investigated in the silenced plants.

**FIGURE 6:**
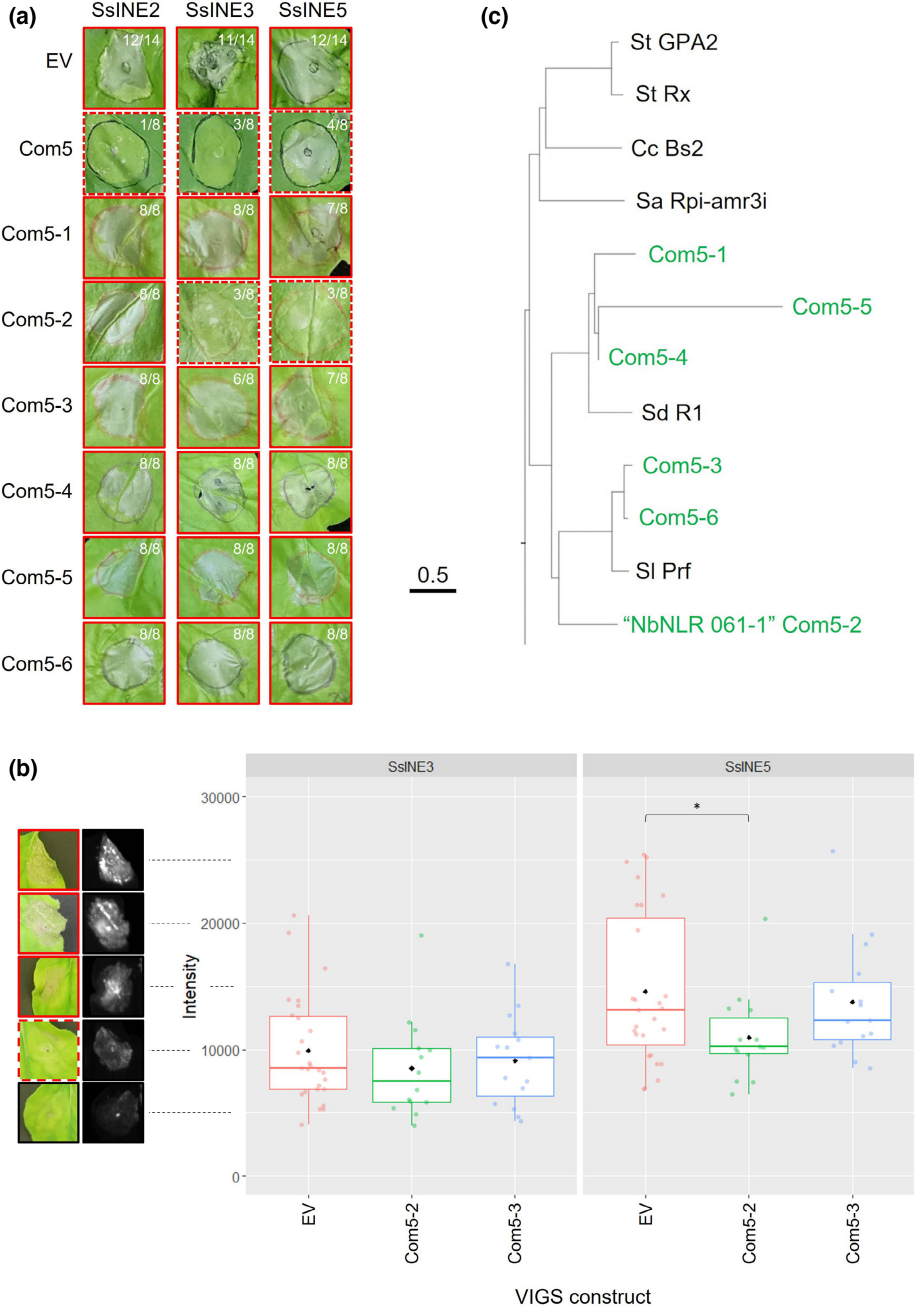
NLR virus‐induced gene silencing (VIGS) screen of SsINE‐induced cell death. (a) Macroscopic cell death symptoms induced by agroinfiltration of SsINE2, SsINE3, and SsINE5 in com5 VIGS *Nicotiana benthamiana* plants. Photographs were taken at 7 days postinfiltration. A red border indicates cell death symptoms; a dashed red border indicates attenuated cell death. The number of replicates with cell death is indicated in the top right corner of each image. (b) Quantification of SsINE3‐ and SsINE5‐induced cell death in NLR gene‐silenced *N. benthamiana* plants. Boxplot showing red light fluorescence of agroinfiltrated *N. benthamiana* leaf sections. Black diamonds represent mean values. Coloured dots represent individual biological replicates. The asterisk (*) indicates a significant difference from the empty vector (EV) negative control (*p* ≤ 0.05), as determined by a Student's *t* test. The photographs and fluorescence images on the left‐hand side are representative images of leaves with fluorescence intensity in approximate increments of 5000. A red border indicates cell death symptoms; a dashed red border indicates weak cell death; a black border indicates no cell death symptoms. (c) Section of a phylogenetic tree showing the relationship between the NLR proteins targeted by the six com5 cassettes and 121 known NLR proteins. Alignment of full‐length amino acid sequences was performed with ClustalW using the Jukes–Cantor model. The neighbour‐joining method was used to construct the phylogenetic tree. The NLR targeted by com5‐2 is labelled as “NbNLR 061‐1.”

This was carried out in an independent laboratory to the initial study. SsINE1, SsINE2, SsINE3, and SsINE5 also induced robust cell death in assays conducted in the independent laboratory; however, SsINE4 did not (Figure S6). Therefore, SsINE4 was excluded from the VIGS assay. In our preliminary screen, we tested the requirement of NbNLRs described in Ahn et al. (2022) for cell death induced by the four aforementioned SsINEs. We found evidence that expression of VIGS cassette com5 reduced cell death induced by SsINE2, SsINE3, and SsINE5 (Figure 6a). The com5 cassette comprises six fragments, each targeting different NLRs.

Following on from this result, extended fragments targeting each of the six NLRs were cloned and expressed (com5‐1 to com5‐6) and then SsINE2, SsINE3, and SsINE5 were expressed in leaf tissue of the silenced *N. benthamiana* plants. As expected, the three SsINEs induced robust cell death in plants agroinfiltrated with an empty silencing vector. Surprisingly, SsINE2 induced cell death in all biological replicates of plants silencing each of the six target NLRs. SsINE3‐ and SsINE5‐induced cell death was attenuated in com5‐2 VIGS plants (five out of eight biological replicates for both effectors; Figure 6a). The NLR targeted by com5‐2 was, therefore, a strong candidate to be mediating cell death induced by SsINE3 and SsINE5.

To confirm these findings, VIGS assays were repeated in an independent laboratory. The *N. benthamiana* phytoene desaturase gene (*NbPDS*) was silenced as a positive silencing control in both assays; the bleaching phenotype indicated successful silencing of the target transcript (Figure S7) (Liu & Page, 2008). In the first assay, com5‐2 and com5‐3 were expressed and then SsINE3 and SsINE5 were expressed. Cell death induced by SsINE5 in com5‐2 VIGS plants was significantly lower than cell death induced by SsINE5 in empty vector control plants, as determined by red light fluorescence quantification (Figure 6b). Cell death induced by SsINE5 was not significantly reduced in com5‐3 VIGS plants and cell death induced by SsINE3 was not significantly reduced in com5‐2 or com5‐3 VIGS plants (Figure 6b). In a further experiment, SsINE3 and SsINE5 were expressed in com5‐2 VIGS plants. Again, cell death induced by SsINE5 but not SsINE3 was reduced in com5‐2 VIGS plants (Figure S8).

The amino acid sequences encoded by the NLR genes targeted by the six com5 cassettes were aligned to 121 known NLR sequences with ClustalW using the Jukes–Cantor model. The neighbour‐joining method was used to construct a phylogenetic tree, which showed that the NLRs targeted by com5‐1, com5‐4, and com5‐5 cluster with *Solanum demissum* potato late blight resistance protein R1 and the NLRs targeted by com5‐2, com5‐3, and com5‐6 cluster with *Solanum lycopersicum* bacterial speck resistance protein Prf (Figure 6c). The two NLR candidates that are targeted by com5‐2 and com5‐3 share 39% and 77% amino acid identity with SlPrf, respectively. Both NLR candidates also share amino acid identity with SdR1 (37% and 26%, respectively). The NLR gene silenced by com5‐2 encodes NbNLR 061‐1, which has an N‐terminal coiled‐coil domain (CNL‐type NLR) (Ahn et al., 2022).

Notably, a BLASTP search revealed that NbNLR 061‐1 has homologues in several plant families across the asterid clade of dicots. The following cut‐offs were used: an E‐value of 1e−5, a query cover of 50%, and 35% amino acid sequence identity (Choudhuri, 2014). There are a total of 57 species that harbour NbNLR 061‐1 homologues, with around half of them (29) in the Solanaceae family (Table S3). These data suggest that SsINE5‐induced cell death in *N. benthamiana* is at least partially dependent on the CNL 061‐1, which has homologues in numerous species in the asterid clade of dicots.

## 3 DISCUSSION

This study has identified five *S. sclerotiorum* necrosis‐inducing effectors, and characterization of the effectors revealed some novel insights into virulence mechanisms employed by *S. sclerotiorum*. We show the first evidence that a broad host range plant pathogen uses an RxLR‐like effector to induce cell death and provide preliminary evidence that an effector may induce cell death via a host NLR.

Previous studies have demonstrated that all SsINEs except SsINE4 are up‐regulated during infection of host species. SsINE1 and SsINE5 were up‐regulated during infection of *B. napus* and *G. max* (soybean), SsINE2 was up‐regulated during infection of *B. napus* and *A. thaliana*, and SsINE3 was up‐regulated during infection of *Cicer arietinum* (chickpea) (Derbyshire et al., 2017; Kusch et al., 2022; Mwape et al., 2021; Seifbarghi et al., 2020; Westrick et al., 2019). Up‐regulation of these genes combined with their effector‐like characteristics points towards a role in virulence. Up‐regulation is host‐dependent, indicating that *S. sclerotiorum* may tailor its effector arsenal to the host species that it encounters. Indeed, *S. sclerotiorum* effector candidates are differentially expressed on different host species (Guyon et al., 2014; Kusch et al., 2022).

SsINE2, encoded by *Sscle05g046060*, has been assayed for necrosis‐inducing activity in a previous study (Seifbarghi et al., 2020). The authors did not observe necrosis after agroinfiltration of SsINE2 with or without an SP. In our study, SsINE2 induced a relatively weak necrosis response. The difference in results could be attributed to the different expression vectors used. SsINE5, encoded by *SS1G_06068*/*Sscle05g045060*, has also been previously characterized. SsINE5, designated as SsSSP3 by Denton‐Giles et al. (2020), was identified as a homologue of a large number of effector candidates in the camellia flower blight pathogen, *Ciborinia camelliae*. Infiltration of SsINE5 protein into camellia petals and *N. benthamiana* leaf tissue was shown to induce necrosis (Denton‐Giles et al., 2020). We were able to replicate the necrosis‐inducing activity in *N. benthamiana* using an agroinfiltration approach and provide evidence that SsINE5 at least partially induces host cell death via an NLR protein. It would be worth investigating whether the cell death caused in camellia petals is mediated by a host immune receptor. The camellia genotype used was Nicky Crisp (*Camellia japonica* × *Camellia pitardii* var. *pitardii*) (Denton‐Giles et al., 2020). Our BLASTP search identified a homologue of the NbNLR involved in mediating SsINE5‐induced necrosis in *Camellia sinensis*. Further investigation into the presence of a homologue in other species within the *Camellia* genus is warranted.

All five GFP‐tagged SsINEs localized at least partially to the nucleus in *N. benthamiana* epidermal cells. SsINE1, SsINE2, and SsINE5 showed particularly strong signals in nuclei. Six previously identified *S. sclerotiorum* necrosis‐inducing effectors also localized at least partially to the nucleus in *N. benthamiana* epidermal cells when expressed both with and without SPs (Seifbarghi et al., 2020). Localization of oomycete RxLR effectors to the nucleus is common. Out of 49 *Hyaloperonospora arabidopsidis* RxLR effector candidates and out of 76 *Plasmopara viticola* RxLR effector candidates, 32 (66%) and 63 (83%), respectively, localized to the nucleus (Caillaud et al., 2012; Liu et al., 2018). In fact, nuclear targeting has been demonstrated for a large number of characterized effectors from all classes of pathogens and nuclear localization is required for the necrosis‐inducing activity of many effector proteins (Deslandes et al., 2003; Rivas & Genin, 2011; Schornack et al., 2010; Yin et al., 2022). In agreement with the nucleus being an important virulence target of pathogen effectors, nuclear localization of host immune receptor NLR proteins is widespread to allow detection of effectors and rapid transcriptional reprogramming to fend off invading pathogens (Shen & Schulze‐Lefert, 2007).

In addition to nuclear localization, SsINE2 and SsINE5 were also observed in small punctate structures. The size of these punctae is consistent with mitochondrial size in green plants, which is generally 0.2–1.5 μm, although without mitochondrial marker colocalization we cannot be certain (Jaipargas et al., 2015). Mitochondria play a key role in governing programmed cell death in plant and animal cells (Vianello et al., 2007). *Salmonella* and *Shigella* bacterial pathogens produce type III secreted effectors that target animal cell mitochondria, resulting in promotion or inhibition of cell death (Nandi et al., 2021). Many of these effectors lack canonical mitochondrial transit peptides, which is also the case for SsINE2 and SsINE5, highlighting the importance of experimental validation of protein subcellular localization. However, the WoLF PSORT prediction for SsINE2 and SsINE5 was apparently consistent with the experimental evidence. Harpin is an elicitor produced by bacterial pathogens *Erwinia amylovora* and *Pseudomonas syringae* that targets mitochondria to induce plant cell death through inhibition of ATP synthesis and rapid cytochrome *c* release (Krause & Durner, 2004; Xie & Chen, 2000). Similarly, the *P. syringae* type III effector AvrRpt2 affects mitochondrial function to induce cell death (Yao et al., 2004). The targeting of *S. sclerotiorum* effectors to mitochondria may represent an effective mechanism to activate cell death in a broad range of host species. Previously, the *S. sclerotiorum* effector SsSSVP1 was shown to interact with a highly conserved subunit of the mitochondrial respiratory chain, QCR8. SsSSVP1 did not directly target the mitochondria but interacted with QCR8 in the cytoplasm and disrupted its correct localization to mitochondria, thereby inducing host cell death (Lyu et al., 2016).

SsINE3 appeared to target chloroplast outer membranes. The *S. sclerotiorum* effector SsITL localizes to host cell chloroplasts, where it interacts with a chloroplast‐localized calcium‐sensing receptor, CAS. This interaction results in reduced salicylic acid accumulation and impaired *S. sclerotiorum* resistance (Tang et al., 2020). The necrosis‐inducing effector ToxA produced by the necrotrophic wheat pathogen *Pyrenophora tritici‐repentis* localizes to chloroplasts in susceptible wheat lines and interacts with a chloroplast‐localized protein named ToxA‐binding protein 1 (ToxABP1) (Manning et al., 2007; Manning & Ciuffetti, 2005). The localization pattern of SsINE3 observed in this study is reminiscent of the PtrToxA localization reported by Manning and Ciuffetti (2005). Neither ToxA nor SsINE3 possesses a canonical chloroplast transit peptide; however, WoLF PSORT predicted chloroplast localization of the mature SsINE3 protein.

Export of the SsINEs to the apoplast reduced or abolished cell death activity. We hypothesize that during infection the conditions created by *S. sclerotiorum* enable host cell entry of effectors. Oxalic acid has been shown to affect cell membrane integrity, which could allow for the translocation of effector proteins into host cells (Tu, 1989).

The SsINEs were highly conserved in globally sourced *S. sclerotiorum* isolates. Their conservation suggests that there may be a high fitness cost to *S. sclerotiorum* if the effectors are lost or nonfunctional. If *S. sclerotiorum* isolates lacking genes encoding functional SsINEs are impaired in infection of host species, this would be expected to affect survival and proliferation of the isolate. Another notable feature of the SsINE proteins is the lack of predicted domains. In addition to predicted SPs, an InterProScan classified SsINE4 into a membrane magnesium transporter InterPro family (MMgT). The absence of conserved domains in the other SsINE proteins is unsurprising given the low amino acid sequence conservation across most effector proteins (Jones et al., 2018).

It was an interesting finding to uncover a functional RxLR‐like motif, defined as [R/K/H]x[L/M/I/F/Y/W]x, in SsINE1 (Kale, 2012; Kale et al., 2010). The RxLR motif is a conserved sequence in intracellular oomycete effectors. We found that the SsINE1 RxLR‐like motif was required for host cell entry in *N. benthamiana* epidermal cells. Effectors with functional RxLR‐like motifs have been identified in the narrow host range fungal pathogens *M. lini*, *F. oxysporum* f. sp. *lycopersici*, and *L. maculans*, but never in a broad host range fungal pathogen (Kale, 2012; Kale et al., 2010; Rafiqi et al., 2010). This opens up the question as to whether SsINE1 requires the RxLR‐like motif to induce cell death in multiple host species. Despite the evidence in the literature that RxLR motifs mediate host cell translocation, Wawra et al. (2017) showed that the RxLR motif of the *P. infestans* effector AVR3a is in fact cleaved before it is secreted by the pathogen. The RxLR motif bears similarity to *Plasmodium* export element (PEXEL) and *Toxoplasma* export element (TEXEL) motifs in effectors of the apicomplexan parasites *Plasmodium falciparum* and *Toxoplasma gondii*, respectively (Hofmann, 2017). These are cleaved prior to secretion in a similar manner to AVR3a to direct the effectors for secretion (Coffey et al., 2016). Whether this is a general process for RxLR and RxLR‐like effectors remains to be seen. Our experiments indicate that the *S. sclerotiorum* SsINE1 RxLR‐like motif plays a role in entry into *N. benthamiana* epidermal cells; however, further experiments would be required to clarify whether the RxLR‐like motif is cleaved during infection or whether it mediates host cell entry in colonized host tissue. Although we identified a dEER‐like motif, we showed that this is not essential for translocation into host cells. A dEER‐like motif has never been found to be required for RxLR‐like fungal effector translocation into host cells. RxLR‐like motifs were also identified in SsINE2, SsINE3, and several of the nonnecrosis‐inducing effectors; however, experimental validation is necessary to determine whether these motifs play a role in effector secretion or translocation into host cells.

The hijacking of effector‐triggered immunity by a necrotrophic effector has not been explicitly demonstrated in a broad host range necrotrophic fungal pathogen. We provide evidence that SsINE5 may induce necrosis dependent on the *NbNLR 061‐1* gene. Cell death was not fully abolished in the silenced plants. This could be due to inefficiency of VIGS; therefore, the silencing efficiency should be quantified to investigate this. In addition, SsINE5 may induce cell death through interaction with more than one virulence target. If this is the case, SsINE5 may depend on NbNLR 061‐1 for strong cell death induction, but it is able to induce attenuated necrosis through other pathways. Further evidence is required to determine explicitly if SsINE5 interacts with NbNLR 061‐1, either directly or indirectly, to activate immunity resulting in programmed cell death. The CNL 061‐1 has homology to thebacterial speck resistance gene of tomato, *Prf*, which encodes a protein that recognizes the *P. syringae* effectors AvrPto and AvrPtoB (Salmeron et al., 1996). A BLASTP search revealed that homologues of the 061‐1 NLR protein exist across the asterid clade of dicots, with around half of them within the Solanaceae family. Investigation of the necrosis‐inducing activity of SsINE5 on these host species and the requirement of the NbNLR 061‐1 homologues for cell death induction would be an interesting avenue of research. We speculate that *S. sclerotiorum* may use SsINE5 to induce cell death on dicotyledonous species carrying a homologue of the *NbNLR 061‐1* gene. If homologues in economically important crop species contribute to susceptibility to *S. sclerotiorum*, they could be removed through traditional breeding or gene editing to enhance resistance to *S. sclerotiorum*.

Cell death induced by the effector SsINE2 was reduced when the com5 VIGS cassette was expressed. This cassette targets six different NbNLRs. However, SsINE2‐induced cell death was not impaired in leaf tissue with each of the six NLRs independently silenced. The com5 targets include genes targeted by com5‐3 and com5‐6 that share 90% amino acid sequence identity. We speculate that these NLRs may function redundantly to recognize SsINE2 and, therefore, the reduction in cell death is only observed when both genes are silenced by the com5 VIGS cassette.

In agreement with our finding that an NLR gene may be required for cell death induction by a *S. sclerotiorum* effector, the *A. thaliana* NLR LAZ5 has been shown to contribute to *S. sclerotiorum* susceptibility (Barbacci et al., 2020). It remains to be seen whether an as yet unknown *S. sclerotiorum* effector activates LAZ5‐triggered immunity in *A. thaliana*; however, this is hypothesized to be the case. Furthermore, Seifbarghi et al. (2020) demonstrated that five necrosis‐inducing effectors require the coreceptor‐like kinases Brassinosteroid Insensitive 1‐Associated Receptor Kinase 1 (BAK1) and Suppressor of BAK1‐Interacting Receptor‐like Kinase 1 (SOBIR1) for cell death induction, indicating that these *S. sclerotiorum* effectors target extracellular immune receptors to activate programmed cell death. Overall, evidence is emerging that *S. sclerotiorum* may hijack the host immune system via extra‐ and intracellular receptors to induce cell death, a favourable outcome for the necrotrophic lifestyle of this broad host range pathogen.

The role of SsINEs in *S. sclerotiorum* virulence should be investigated through the development of knockout or knockdown strains followed by phenotyping assays to test for a reduction in pathogenicity. It is likely that there is some functional redundancy among necrosis‐inducing proteins, and therefore an absence of phenotypic change in knockout or knockdown strains would not necessarily indicate that the gene is not involved in the infection process. In addition, future work should test secretion from *S. sclerotiorum* during infection and subsequent localization within host tissue to validate the findings from SsINE overexpression in *N. benthamiana* by agroinfiltration.

In summary, through identification and characterization of five necrosis‐inducing effectors in *S. sclerotiorum*, we have furthered our understanding of how a broad host range fungal pathogen induces host cell death. The evidence presented in this study illustrates that an effector of a broad host range pathogen uses an RxLR‐like motif to translocate into host cells and another effector may hijack the host immune system via a clade‐specific NLR to trigger necrosis. Elucidation of the molecular mechanisms of pathogenicity uncovers opportunities for novel approaches to control damaging disease caused by *S. sclerotiorum*, such as through removal of host susceptibility genes.

## 4 EXPERIMENTAL PROCEDURES

### 4.1 Plant growth

In experiments conducted at Curtin University, *N. benthamiana* plants were grown in 0.5‐L pots filled with UWA plant biology potting mix (Richgro). Plants were grown in a controlled environment growth chamber with a photoperiod of 16 h light and 8 h of darkness, a temperature of 22°C, and a relative humidity of 45%. Plants were fertilized with Nitrophoska Perfect after 3 weeks of growth.

In experiments conducted at POSTECH, *N. benthamiana* plants were grown in Gyeongju Chemical mix (Dong‐sin). Plants were grown in a plant growth room with a photoperiod of 11 h light per day and a temperature of 22°C.

### 4.2 Bioinformatics analysis

The localization of 70 effector candidates identified in the reference *S. sclerotiorum* isolate 1980 was predicted using ApoplastP v. 1.0 (Derbyshire et al., 2017; Sperschneider et al., 2018). Using Geneious Prime v. 2022.1.1, the sequences of the 24 putative cytoplasmic effector genes in isolate 1980 were aligned to a genome sequence of the aggressive Australian isolate CU8.24 to identify the corresponding effector genes in isolate CU8.24 (Kearse et al., 2012). The subcellular localization of the set of putative cytoplasmic effectors in isolate CU8.24 was then predicted using LOCALIZER v. 1.0.4 and WoLF PSORT (Horton et al., 2007; Sperschneider et al., 2017). For WoLF PSORT prediction, we used mature effector proteins in plant mode. The localizations predicted are those found in at least five of the 14 reference proteins reported by WoLF PSORT, as conducted by Robin et al. (2018). SignalP v. 4.1 was used to predict SPs (Petersen et al., 2011).

InterProScan was used to identify conserved protein domains in the amino acid sequences of SsINEs (Zdobnov & Apweiler, 2001). Amino acid sequences of SsINEs were compared in 26 *S. sclerotiorum* isolates using previously generated genome sequences (Derbyshire et al., 2019). These isolates were sourced from Australia (12), Europe (5), North America (7), and Africa (2). The isolates were collected primarily from *B. napus* (19) but also from *Lupinus* spp. (lupin) (3), *Trifolium* sp. (clover) (1), *Carthamus tinctorius* (safflower) (1), and *Phaseolus vulgaris* (common bean) (1). The host species is unknown for one isolate (Table S1) (Amselem et al., 2011; Derbyshire et al., 2019). Firstly, CU8.24 *SsINE* gene sequences were aligned to the genome sequences and gene sequences from each isolate were extracted. The coding sequences were translated to amino acids and aligned by Geneious alignment with default settings. Figure 3a alignments were made using T‐Coffee (https://tcoffee.crg.eu/apps/tcoffee/do:regular) and Boxshade (http://www.ch.embnet.org/software/BOX_form.html).

### 4.3 Gene cloning and construct generation

Effector coding sequences without predicted SPs were amplified using gene‐specific primers with partial attB sites. Single‐exon genes were amplified from CU8.24 genomic DNA, whereas multi‐exon genes were amplified from cDNA derived from bulked in vitro and in planta (1 h postinoculation [HPI], 6 HPI, 24 HPI, and 48 HPI) mycelia samples. Full attB sites were introduced by primers in a second round of amplification. The attB‐tailed PCR products were cloned into the entry vector Invitrogen Gateway pDONR/Zeo by BP recombination (Invitrogen). LR recombination was then carried out to transfer the coding sequences from pDONR/Zeo to the *Agrobacterium*‐mediated transient expression destination vectors pEAQ‐HT‐DEST1‐GFP and pEAQ‐HT‐DEST1‐MtPR1SP‐GFP derived from the pEAQ vectors developed by Sainsbury et al. (2009). These vectors harbour the constitutive cauliflower mosaic virus (CaMV) 35S promoter to drive expression of the effectors.

To introduce specific mutations, the Q5 site‐directed mutagenesis kit (New England Biolabs) was used according to the manufacturer's protocol. pTRV2 silencing constructs were generated by amplifying target gene fragments from *N. benthamiana* genomic DNA and assembling into the GoldenGate‐compatible pTRV2 vector using GoldenGate assembly (Choi et al., 2021). All constructs and primers are listed in Table S4 and Table S5.

### 4.4 *Agrobacterium*‐mediated transient expression (agroinfiltration) in *N. benthamiana*

The generated expression vectors were transformed into *Agrobacterium tumefaciens* AGL1 by electroporation. The transformed *A. tumefaciens* strains were cultured on Luria–Bertani agar supplemented with rifampicin and kanamycin and then cultured in Luria–Bertani broth supplemented with rifampicin and kanamycin for 16–24 h. Cells were centrifuged at 3300 × *g* for 4 min, washed in 1 mL infiltration medium (10 mM MgCl_2_, 10 mM MES, pH 5.6), centrifuged once more, and then suspended in 2 mL infiltration medium. Suspensions were then adjusted to OD_600_ = 0.6 and 200 μM of acetosyringone was added. Bacterial suspensions were incubated at room temperature for 2–4 h with gentle shaking and then infiltrated into the abaxial leaf surfaces of 4–6‐week‐old *N. benthamiana* plants using a blunt‐end syringe. Cell death was observed and photographed at 7 days postinfiltration (DPI). Experiments were conducted at least three times with similar results.

### 4.5 Western blotting

*A. tumefaciens* strains carrying respective expression vectors were agroinfiltrated in *N. benthamiana* leaves. Leaves were sampled at 2 DPI and flash frozen in liquid nitrogen. Two leaves per strain were ground in liquid nitrogen and total proteins were extracted from 2 g of tissue in 2 mL of protein extraction buffer (20 mM Tris–HCl pH 7.5, 300 mM NaCl, 5 mM MgCl_2_) supplemented with 0.5% (vol/vol) IGEPAL CA‐630, 5 mM dithiothreitol, and one tablet of cOmplete Protease Inhibitor Cocktail (Roche) per 50 mL. Samples were centrifuged at 3200 × *g* at 4°C for 15 min. Supernatants were filtered through two layers of Miracloth (Millipore) and used as total protein extract samples. Proteins were separated by SDS‐PAGE and analysed by immunoblotting with an anti‐GFP primary antibody (Thermo Fisher) and a horseradish peroxidase‐conjugated goat anti‐rabbit secondary antibody (Agrisera). Proteins were detected using Pierce ECL western blotting substrate (Thermo Fisher). Polyvinylidene difluoride (PVDF) membranes were stained with Ponceau S (Sigma) to visualize protein loading.

### 4.6 Cell death quantification assays

For the ion leakage assay, the protocol of Yu et al. (2012) was followed with some modifications. Three leaf discs were taken per agroinfiltrated zone at 5 DPI and floated on 2 mL water in a 12‐well plate. This represented one biological replicate. Three biological replicates were taken per treatment. Conductivity was measured using a Horiba EC‐11 LAQUAtwin compact conductivity meter (Horiba). This experiment was conducted twice with similar results. Analysis of variance was conducted with a post hoc Tukey honestly significant difference test to determine significant differences between relative ion leakage values.

To quantify cell death using red light fluorescence, agroinfiltrated *N. benthamiana* leaves were sampled at 7 DPI and analysed using a ChemiDoc MP imaging system, model Universal Hood III (Bio‐Rad) according to the protocol developed by Landeo Villanueva et al. (2021). To detect red light fluorescence, the exposure time was set to 2–4 s (the same exposure time was used for all samples within an experiment). To detect GFP fluorescence, the exposure time was set to 0.1–0.3 s (the same exposure time was used for all samples within an experiment). For the SsINE1 variant cell death assay (Figure 4c), 16 infiltrated zones were analysed per treatment. This experiment was conducted three times with similar results. For the first NLR VIGS assay (Figure 6b), 14–27 infiltrated zones were analysed per treatment; for the second NLR VIGS assay (Figure S8), 45–47 infiltrated zones were analysed per treatment. Student's *t* tests were used to determine significant differences from control treatments.

### 4.7 Confocal microscopy

Agroinfiltrated *N. benthamiana* leaf tissue was sampled at 2–3 DPI, mounted on a microscope slide, and viewed on a Nikon A1+ point scanning confocal microscope (Nikon). To assess apoplastic localization, samples were incubated in 30% glycerol for at least 30 min to induce plasmolysis prior to mounting. GFP fluorescence and chloroplast autofluorescence were excited at 489 nm and emission was captured at 525 nm and 700 nm, respectively. Objective magnification of 10× or 20× was used.

### 4.8 VIGS assay

*N. benthamiana* was grown for approximately 2 weeks until the plants had two to four true leaves. Two leaves were coinfiltrated with *A. tumefaciens* strains transformed with pTRV1 and a pTRV2 construct (OD_600_ = 0.4 for each strain). Successful silencing was confirmed by silencing the *NbPDS* gene and observing the expected bleaching phenotype. After 2 or 3 further weeks of growth, plants were agroinfiltrated with effector expression vectors as described above. The development and use of the NbNLR VIGS library is described in Ahn et al. (2022).

## CONFLICT OF INTEREST STATEMENT

The authors declare that they have no conflict of interest.

## Supporting information


**Figure S1** Necrosis‐inducing activity of SsINE proteins in *Nicotiana benthamiana* leaf tissue with and without a signal peptide. Macroscopic cell death symptoms induced by agroinfiltration of SsINE1–5 with and without a signal peptide (SP and ΔSP, respectively). Green fluorescent protein (GFP) was included as a negative control. Photographs were taken at 7 days postinfiltration. A red border indicates cell death symptoms; a dashed red border indicates attenuated cell death; a black border indicates no cell death symptoms.

**Figure S2** Subcellular localization of SsINE proteins expressed with a signal peptide in *Nicotiana benthamiana* epidermal cells. Green fluorescent protein (GFP) was included as a control. The leaf samples were plasmolysed prior to mounting on microscope slides. Panels on the left‐hand side labelled “GFP” show GFP fluorescence. Panels on the right‐hand side labelled “Merged” show GFP fluorescence, chloroplast autofluorescence, and bright field images. The white arrows indicate nuclei; the red arrows indicate apoplastic localization. Images are *z*‐projections. The scale bars are 50 μm.

**Figure S3** Western blot analysis of SsINE proteins expressed in *Nicotiana benthamiana* leaf tissue by agroinfiltration with and without a signal peptide. SsINE‐GFP fusion proteins were detected by immunoblotting with an anti‐GFP antibody on total protein extracts. Red asterisks indicate the respective proteins. Staining of the PVDF membrane with Ponceau S shows protein loading and transfer (the band shown is the RuBisCO large subunit).

**Figure S4** Western blot analysis of SsINE1 protein variants expressed in *Nicotiana benthamiana* leaf tissue by agroinfiltration with and without a signal peptide. SsINE1‐GFP fusion proteins were detected by immunoblotting with an anti‐GFP antibody on total protein extracts. Red asterisks indicate the respective proteins. Staining of the PVDF membrane with Ponceau S shows protein loading and transfer (the band shown is the RuBisCO large subunit).

**Figure S5** Subcellular localization of SsINE1 variants expressed without a signal peptide in *Nicotiana benthamiana* epidermal cells. Green fluorescent protein (GFP) was included as a control. Panels on the left‐hand side labelled “GFP” show GFP fluorescence. Panels on the right‐hand side labelled “Merged” show GFP fluorescence, chloroplast autofluorescence, and bright field images. The white arrows indicate nuclei; the red arrows indicate apoplastic localization. Images are *z*‐projections. The scale bars are 100 μm.

**Figure S6** Necrosis‐inducing activity of SsINE proteins in *Nicotiana benthamiana* leaf tissue in experiments conducted at POSTECH. Macroscopic cell death symptoms induced by agroinfiltration of SsINE1–5 without a signal peptide (ΔSP). Empty vector was included as a negative control. Photographs were taken at 7 days postinfiltration. A red border indicates cell death symptoms; a black border indicates no cell death symptoms.

**Figure S7** Photographs of 4‐week‐old *Nicotiana benthamiana* plants agroinfiltrated with TRV constructs. The bleaching phenotype of the plants infiltrated with TRV1 and TRV2:*NbPDS* indicates successful silencing of the *NbPDS* gene.

**Figure S8** Quantification of SsINE3‐ and SsINE5‐induced cell death in *NbNLR 061‐1*‐silenced *Nicotiana benthamiana* plants (second independent experiment). Boxplot showing red light fluorescence of agroinfiltrated *N. benthamiana* leaf sections. Black diamonds represent mean values. Coloured dots represent individual biological replicates. The asterisk (*) indicates a significant difference from the empty vector (EV) negative control (*p* ≤ 0.05), as determined by a Student’s *t* test.

**Table S1** SsINE alleles in globally sourced *Sclerotinia sclerotiorum* isolates.

**Table S2** Presence of RxLR‐like motifs in candidate effectors.

**Table S3** Homologues of the NbNLR 061‐1 protein.

**Table S4** Constructs used in this study.

**Table S5** Primers used in this study.

## Data Availability

The data that support the findings of this study are available from the corresponding author upon reasonable request.
