# Supplementary figures and images for: The broad host range pathogen Sclerotinia sclerotiorum produces multiple effector proteins that induce host cell death intracellularly
Source: Mol Plant Pathol. 2023 Apr 10;24(8):866–81. doi: 10.1111/mpp.13333 (PMC10346375; doi:10.1111/mpp.13333)

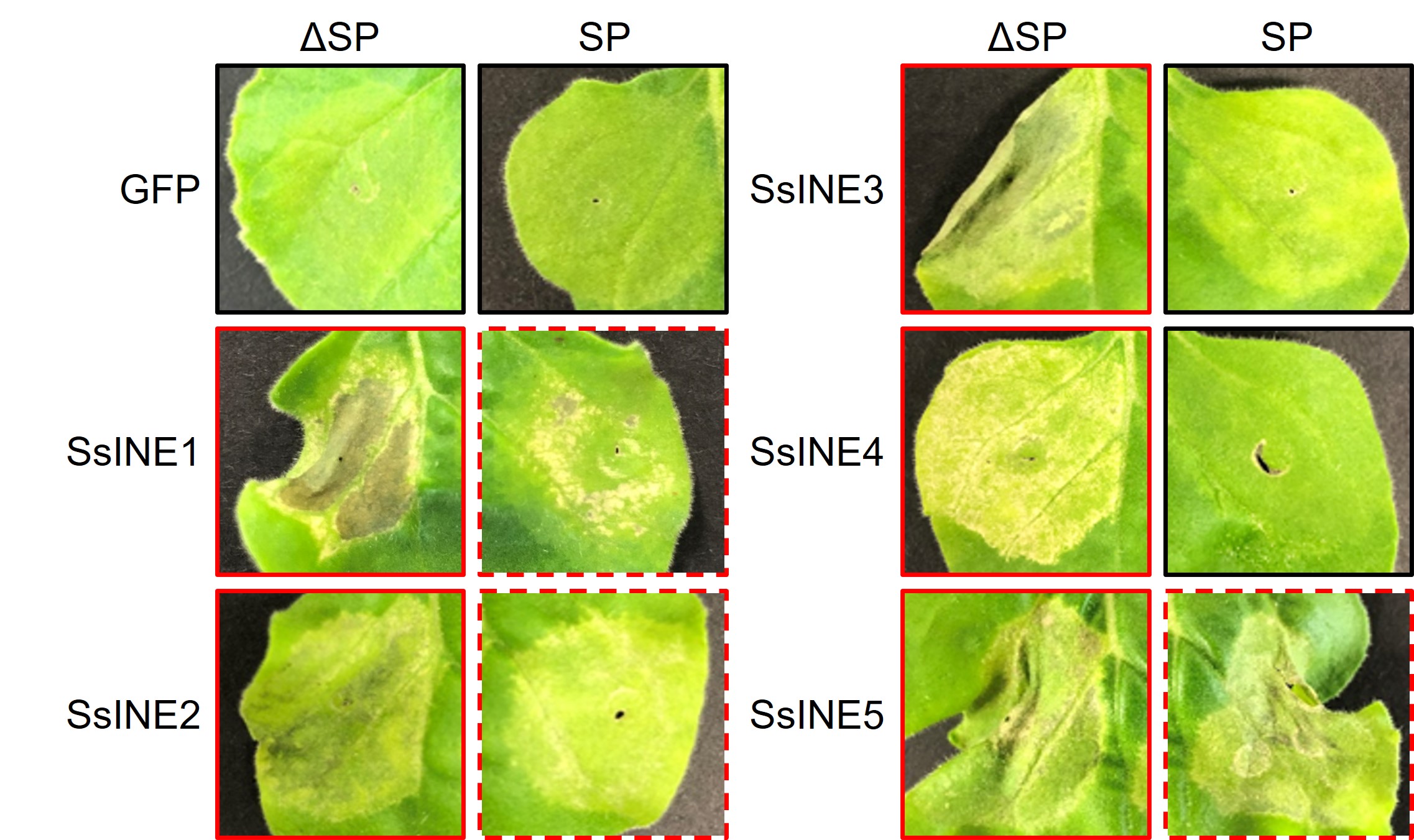

Supplement: Supplementary file 1 — Figure S1 Necrosis‐inducing activity of SsINE proteins in Nicotiana benthamiana leaf tissue with and without a signal peptide. Macroscopic cell death symptoms induced by agroinfiltration of SsINE1–5 with and without a signal peptide (SP and ΔSP, respectively). Green fluorescent protein (GFP) was included as a negative control. Photographs were taken at 7 days postinfiltration. A red border indicates cell death symptoms; a dashed red border indicates attenuated cell death; a black border indicates no cell death symptoms. [file MPP-24-866-s002.jpg]

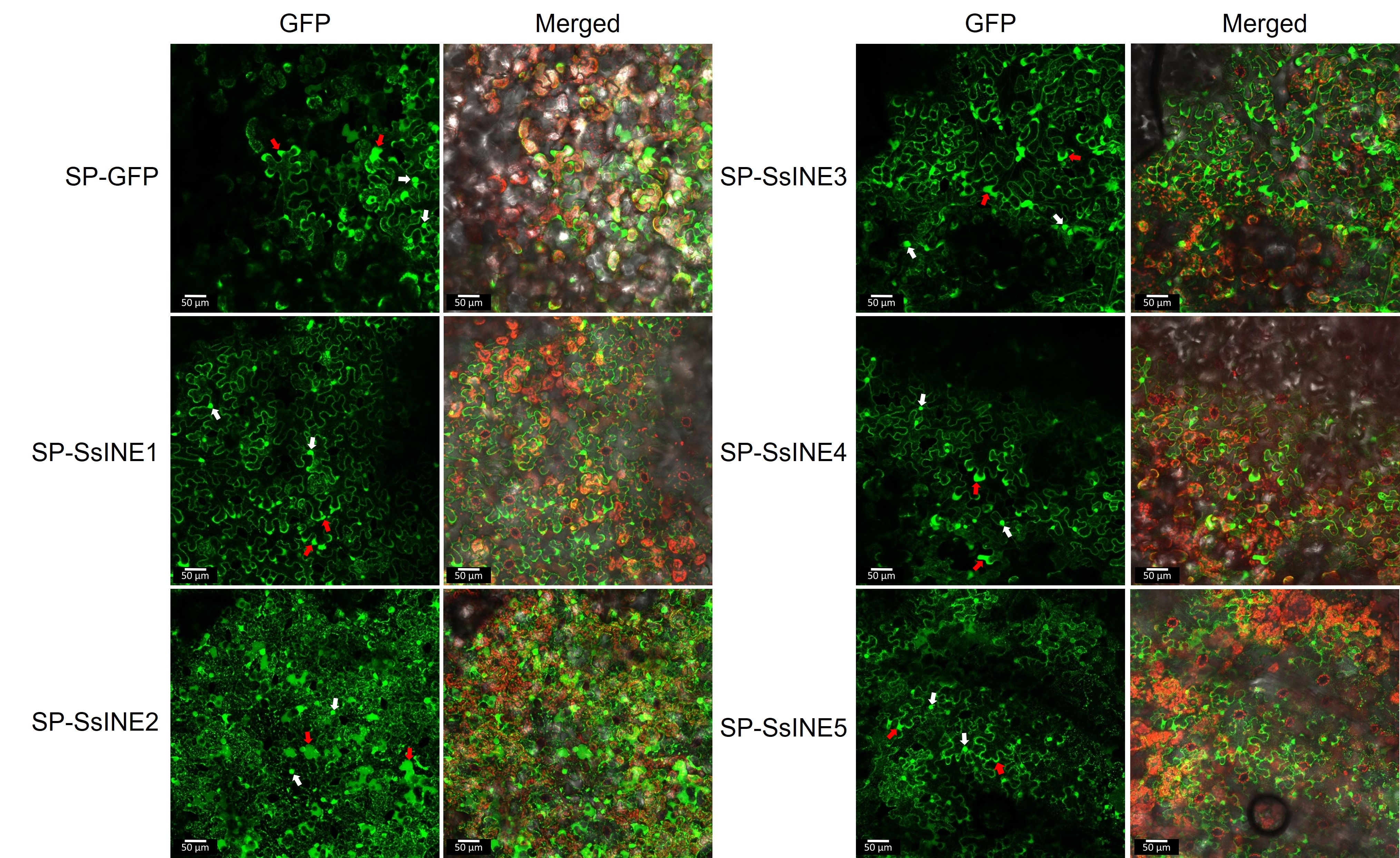

Supplement: Supplementary file 2 — Figure S2 Subcellular localization of SsINE proteins expressed with a signal peptide in Nicotiana benthamiana epidermal cells. Green fluorescent protein (GFP) was included as a control. The leaf samples were plasmolysed prior to mounting on microscope slides. Panels on the left‐hand side labelled “GFP” show GFP fluorescence. Panels on the right‐hand side labelled “Merged” show GFP fluorescence, chloroplast autofluorescence, and bright field images. The white arrows indicate nuclei; the red arrows indicate apoplastic localization. Images are z‐projections. The scale bars are 50 μm. [file MPP-24-866-s005.jpg]

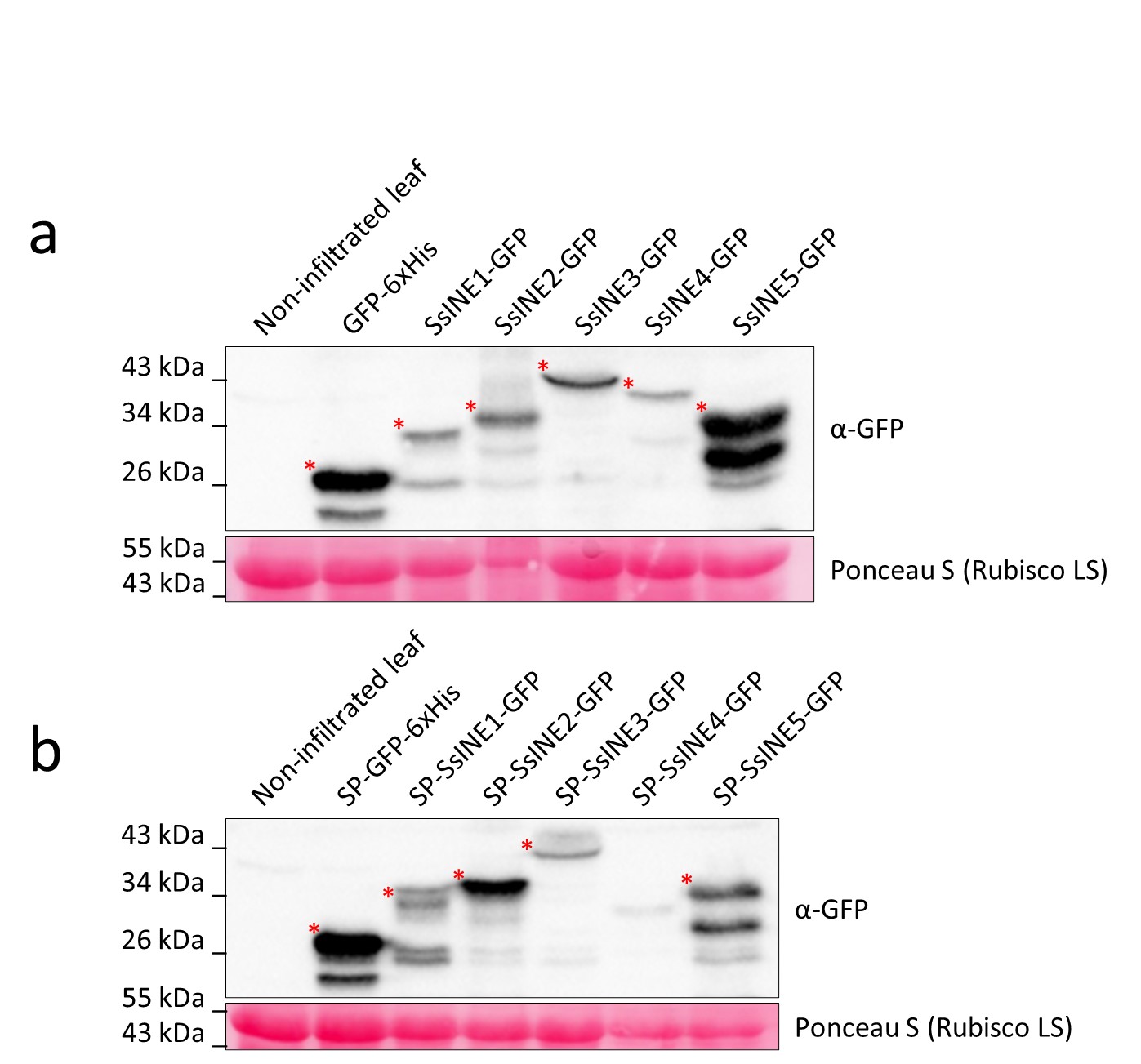

Supplement: Supplementary file 3 — Figure S3 Western blot analysis of SsINE proteins expressed in Nicotiana benthamiana leaf tissue by agroinfiltration with and without a signal peptide. SsINE‐GFP fusion proteins were detected by immunoblotting with an anti‐GFP antibody on total protein extracts. Red asterisks indicate the respective proteins. Staining of the PVDF membrane with Ponceau S shows protein loading and transfer (the band shown is the RuBisCO large subunit). [file MPP-24-866-s010.jpg]

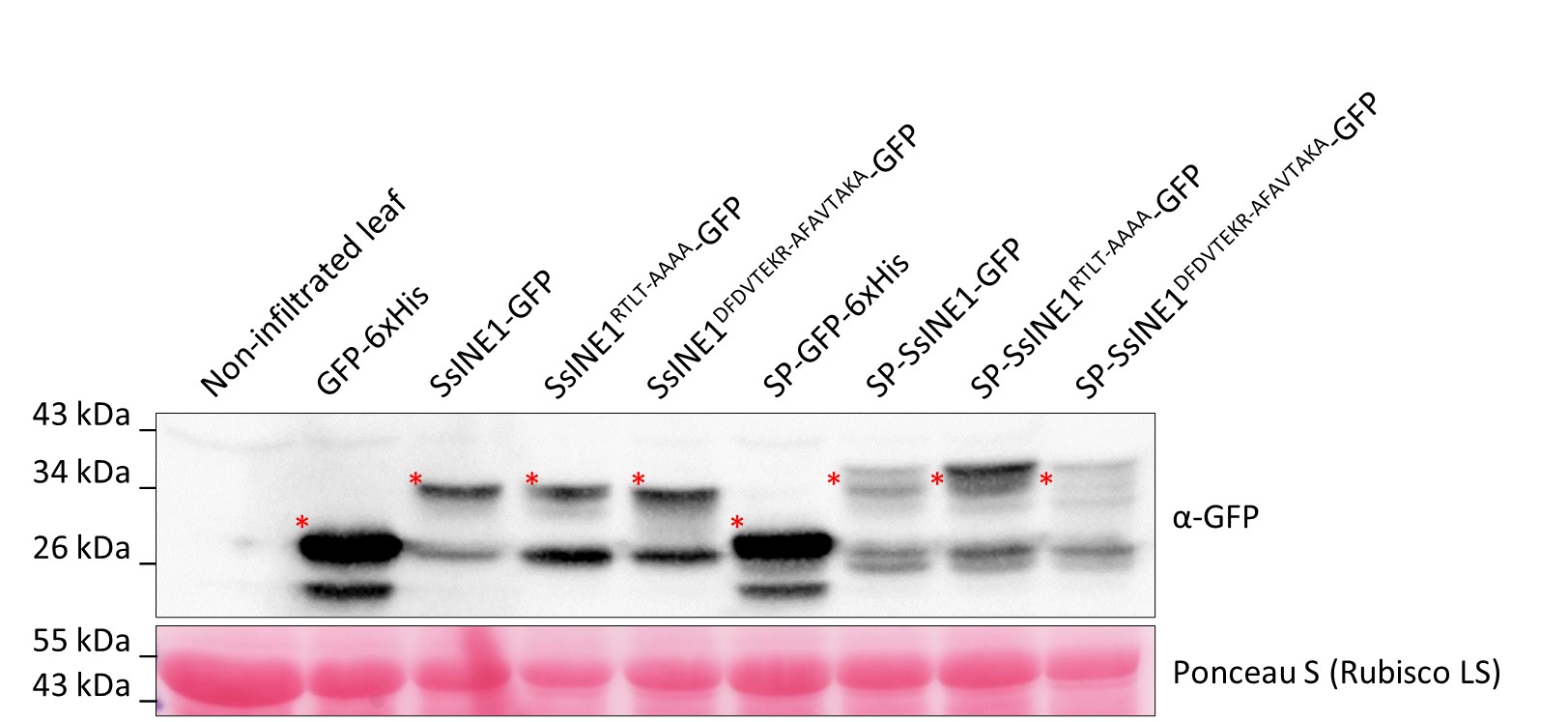

Supplement: Supplementary file 4 — Figure S4 Western blot analysis of SsINE1 protein variants expressed in Nicotiana benthamiana leaf tissue by agroinfiltration with and without a signal peptide. SsINE1‐GFP fusion proteins were detected by immunoblotting with an anti‐GFP antibody on total protein extracts. Red asterisks indicate the respective proteins. Staining of the PVDF membrane with Ponceau S shows protein loading and transfer (the band shown is the RuBisCO large subunit). [file MPP-24-866-s007.jpg]

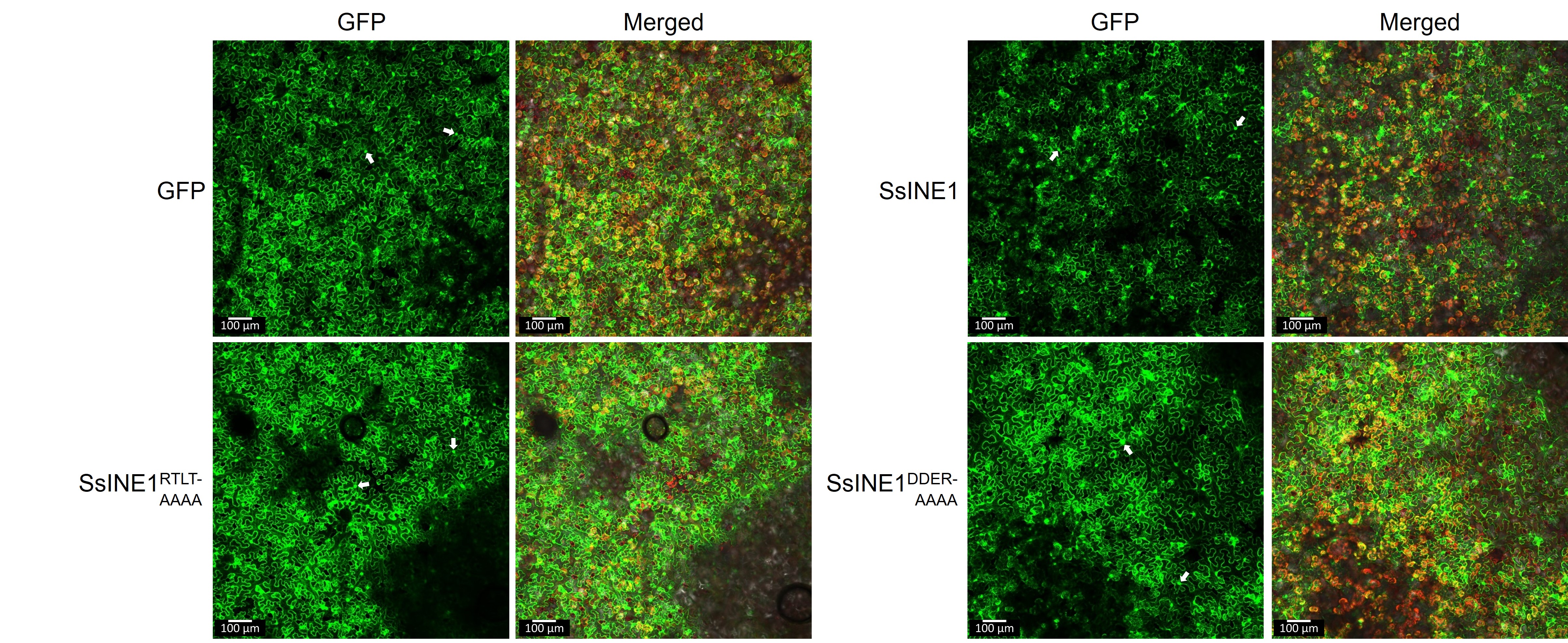

Supplement: Supplementary file 5 — Figure S5 Subcellular localization of SsINE1 variants expressed without a signal peptide in Nicotiana benthamiana epidermal cells. Green fluorescent protein (GFP) was included as a control. Panels on the left‐hand side labelled “GFP” show GFP fluorescence. Panels on the right‐hand side labelled “Merged” show GFP fluorescence, chloroplast autofluorescence, and bright field images. The white arrows indicate nuclei; the red arrows indicate apoplastic localization. Images are z‐projections. The scale bars are 100 μm. [file MPP-24-866-s013.jpg]

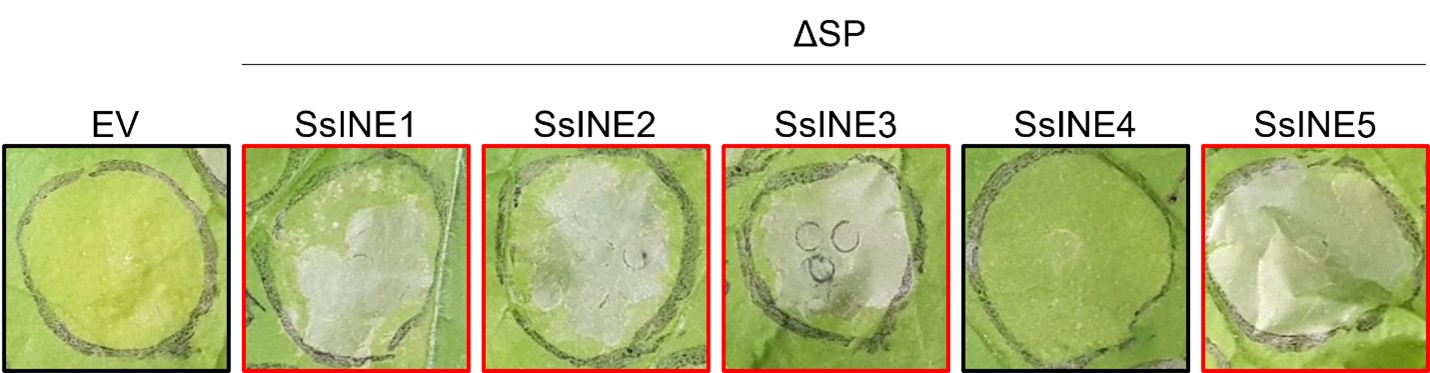

Supplement: Supplementary file 6 — Figure S6 Necrosis‐inducing activity of SsINE proteins in Nicotiana benthamiana leaf tissue in experiments conducted at POSTECH. Macroscopic cell death symptoms induced by agroinfiltration of SsINE1–5 without a signal peptide (ΔSP). Empty vector was included as a negative control. Photographs were taken at 7 days postinfiltration. A red border indicates cell death symptoms; a black border indicates no cell death symptoms. [file MPP-24-866-s006.jpg]

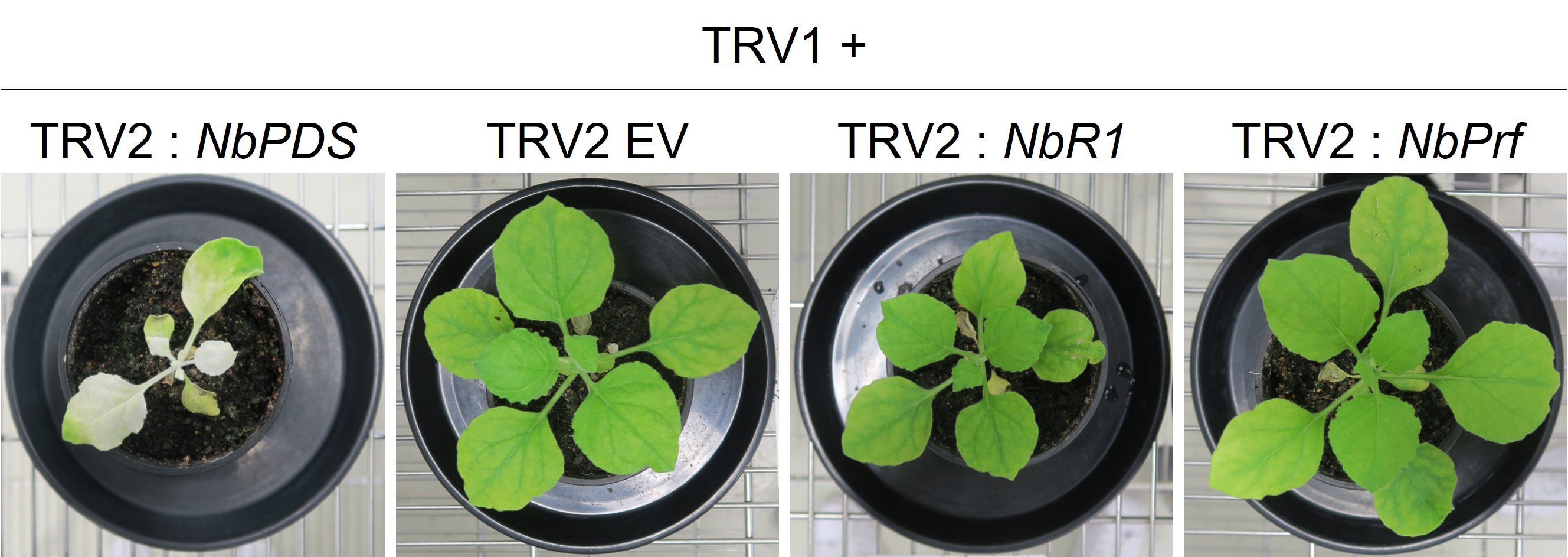

Supplement: Supplementary file 7 — Figure S7 Photographs of 4‐week‐old Nicotiana benthamiana plants agroinfiltrated with TRV constructs. The bleaching phenotype of the plants infiltrated with TRV1 and TRV2:NbPDS indicates successful silencing of the NbPDS gene. [file MPP-24-866-s012.jpg]

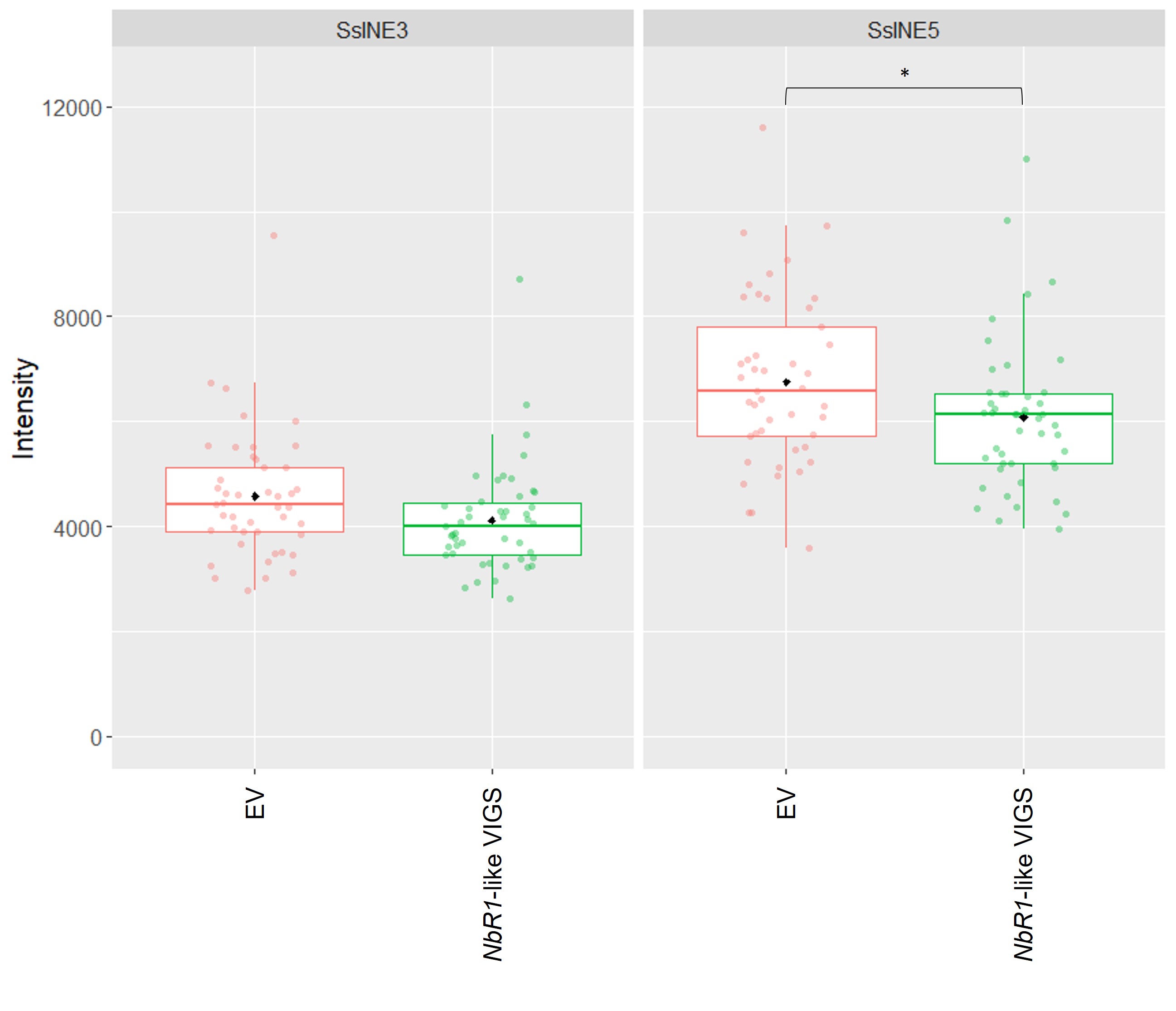

Supplement: Supplementary file 8 — Figure S8 Quantification of SsINE3‐ and SsINE5‐induced cell death in NbNLR 061‐1‐silenced Nicotiana benthamiana plants (second independent experiment). Boxplot showing red light fluorescence of agroinfiltrated N. benthamiana leaf sections. Black diamonds represent mean values. Coloured dots represent individual biological replicates. The asterisk (*) indicates a significant difference from the empty vector (EV) negative control (p ≤ 0.05), as determined by a Student’s t test. [file MPP-24-866-s009.jpg]
