# Supplementary material for: The broad host range pathogen Sclerotinia sclerotiorum produces multiple effector proteins that induce host cell death intracellularly
Source: Mol Plant Pathol. 2023 Apr 10;24(8):866–81. doi: 10.1111/mpp.13333 (PMC10346375; doi:10.1111/mpp.13333)
Supplement: Supplementary file 9 — Table S1 SsINE alleles in globally sourced Sclerotinia sclerotiorum isolates. [file MPP-24-866-s011.docx]

**Table S1** SsINE alleles in globally sourced *S. sclerotiorum* isolates.

| **Isolate** | **Geographic origin** | **Host** | **SsINE1 allele** | **SsINE3 allele** |
| --- | --- | --- | --- | --- |
| 321 | Olds, Alberta | Rapeseed | 1980 | 1980 |
| 1980 | USA | Soybean | 1980 | 1980 |
| AB29 | Cayley, Alberta | Rapeseed | 1980 | 1980 |
| BloC014 | Blois, France | Rapeseed | 1980 | 1980 |
| BloC104 | Blois, France | Rapeseed | 1980 | 1980 |
| CU10.12 | Geraldton, Western Australia, Australia | Rapeseed | 1980 | Sssaf |
| CU10.17 | Geraldton, Western Australia, Australia | Rapeseed | 1980 | 1980 |
| CU10.20 | Geraldton, Western Australia, Australia | Rapeseed | 1980 | 1980 |
| CU10.7 | Geraldton, Western Australia, Australia | Rapeseed | 1980 | 1980 |
| CU11.19 | Geraldton, Western Australia, Australia | Rapeseed | 1980 | 1980 |
| CU11.7 | Eneabba, Western Australia, Australia | Lupin | 1980 | Sssaf |
| CU4.2 | Mount Barker, Western Australia, Australia | Rapeseed | 1980 | 1980 |
| CU6.1 | Mount Barker, Western Australia, Australia | Rapeseed | 1980 | 1980 |
| CU8.24 | South Stirling, Western Australia, Australia | Rapeseed | 1980 | Sssaf |
| CU8.30 | South Stirling, Western Australia, Australia | Rapeseed | 1980 | Sssaf |
| CULa | Unknown, Western Australia, Australia | Lupin | 1980 | Sssaf |
| CULm | Unknown, Western Australia, Australia | Lupin | 1980 | Sssaf |
| FrB5 | Dijon, France | Clover | 1980 | 1980 |
| MB21 | Morton, Manitoba | Rapeseed | 1980 | 1980 |
| MB52 | Shoal Lake, Manitoba | Rapeseed | 1980 | 1980 |
| P163 | Blois, France | Rapeseed | 1980 | 1980 |
| P314 | Blois, France | Rapeseed | 1980 | 1980 |
| S55 | USA | Rapeseed | 1980 | 1980 |
| SK35 | Rouleau, Saskatchewan | Rapeseed | 1980 | 1980 |
| Ss44 | Morocco (Meknès) | Safflower | 1980 | 1980 |
| Sssaf | South Africa | Unknown | Sssaf | Sssaf |
