# Supplementary material for: The broad host range pathogen Sclerotinia sclerotiorum produces multiple effector proteins that induce host cell death intracellularly
Source: Mol Plant Pathol. 2023 Apr 10;24(8):866–81. doi: 10.1111/mpp.13333 (PMC10346375; doi:10.1111/mpp.13333)
Supplement: Supplementary file 10 — Table S2 Presence of RxLR‐like motifs in candidate effectors. [file MPP-24-866-s004.docx]

| **Putative effector ID** | **RxLR-like motif (within N-terminus of mature effector)** | **Amino acid sequence of RxLR-like motif** |
| --- | --- | --- |
| Sscle01g005390 | No | - |
| Sscle01g006330 | No | - |
| Sscle01g009960 | Yes | RGLT |
| Sscle02g012940 (SsINE1) | Yes | RTLT |
| Sscle02g021780 | Yes | KEFD |
| Sscle04g039210 | Yes | KALC |
| Sscle05g045060 (SsINE5) | No | - |
| Sscle05g046060 (SsINE2) | Yes | HKIC |
| Sscle05g046070 | No | - |
| Sscle06g050820 | Yes | KVYT, RDLE |
| Sscle07g057000 | Yes | RLLG, RALS |
| Sscle10g075140 | Yes | RDLE |
| Sscle10g080580 | Yes | RVFI |
| Sscle11g081020 | Yes | KHIK, KFYI |
| Sscle12g087960 (SsINE3) | Yes | HIYD |
| Sscle12g088660 | Yes | KSLS, RHLD |
| Sscle13g094760 (SsINE4) | No | - |
| Sscle13g094920 | Yes | HNIQ, KYLY |
| Sscle13g095230 | No | - |
| Sscle14g100310 | Yes | LQIR, RFLA |
| Sscle16g107890 | No | - |

**Table S2** Presence of RxLR-like motifs in candidate effectors.
