# Supplementary material for: The broad host range pathogen Sclerotinia sclerotiorum produces multiple effector proteins that induce host cell death intracellularly
Source: Mol Plant Pathol. 2023 Apr 10;24(8):866–81. doi: 10.1111/mpp.13333 (PMC10346375; doi:10.1111/mpp.13333)
Supplement: Supplementary file 11 — Table S3 Homologues of the NbNLR 061‐1 protein. [file MPP-24-866-s008.docx]

| **Clade** | **Subclade** | **Order** | **Plant family** | **Number of species** |
| --- | --- | --- | --- | --- |
| Asterids | Lamiids | Solanales | Solanaceae | 29 |
| Asterids | Campanulids | Asterales | Asteraceae | 5 |
| Asterids | Lamiids | Lamiales | Lamiaceae | 4 |
| Asterids | Lamiids | Gentianales | Rubiaceae | 3 |
| Asterids | Lamiids | Lamiales | Oleaceae | 2 |
| Asterids | Lamiids | Solanales | Convolvulaceae | 2 |
| Asterids | NA | Ericales | Actinidiaceae | 2 |
| Asterids | Lamiids | Lamiales | Paulowniaceae | 1 |
| Asterids | Lamiids | Lamiales | Pedialaceae | 1 |
| Asterids | NA | Cornales | Nyssaceae | 1 |
| Asterids | Lamiids | Lamiales | Scrophulariaceae | 1 |
| Asterids | Campanulids | Apiales | Apiaceae | 1 |
| Asterids | NA | Ericales | Theaceae | 1 |
| Asterids | Lamiids | Lamiales | Phrymaceae | 1 |
| Asterids | Lamiids | Lamiales | Gesneriaceae | 1 |
| Asterids | Lamiids | Boraginales | Boriganaceae | 1 |
| Asterids | Lamiids | Lamiales | Orobranchaceae | 1 |
| BLASTP cut-offs: E-value of 1e-5, query cover of 50%, percent identity of 35% | | | |  |
|  | | | |  |

**Table S3** Homologues of the NbNLR 061-1 protein.
